# Competition between CO_2_-philicity and Mixing Entropy Leads to CO_2_ Solubility Maximum in Polyether Polyols

**DOI:** 10.1021/acs.iecr.2c02396

**Published:** 2022-08-18

**Authors:** Andrew S. Ylitalo, Huikuan Chao, Pierre J. Walker, Jacob Crosthwaite, Thomas C. Fitzgibbons, Valeriy G. Ginzburg, Weijun Zhou, Zhen-Gang Wang, Ernesto Di Maio, Julia A. Kornfield

**Affiliations:** † Division of Chemistry and Chemical Engineering, California Institute of Technology, Pasadena, California 91125, United States; ‡ Dow, Inc., Midland, Michigan 48667, United States; ¶ Dow, Inc., Lake Jackson, Texas 77566, United States; § Michigan State University, East Lansing, Michigan 48910, United States; ∥ Dipartimento di Ingegneria Chimica, dei Materiali e della Produzione Industriale, University of Naples, Federico II, P. le Tecchio 80, 80125 Naples, Italy

## Abstract

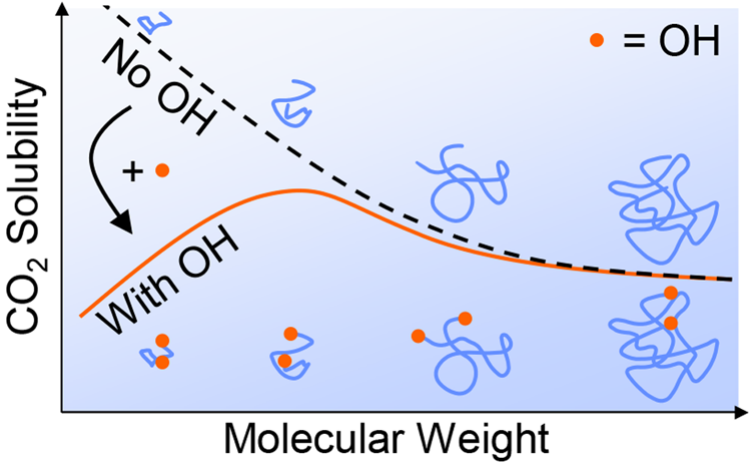

In carbon dioxide-blown polymer foams, the solubility of carbon dioxide (CO_2_) in the polymer profoundly shapes the structure and, consequently, the physical properties of the foam. One such foam is polyurethane—commonly used for thermal insulation, acoustic insulation, and cushioning—which increasingly relies on CO_2_ to replace environmentally harmful blowing agents. Polyurethane is produced through the reaction of isocyanate and polyol, of which the polyol has the higher capacity for dissolving CO_2_. While previous studies have suggested the importance of the effect of hydroxyl end groups on CO_2_ solubility in short polyols (<1000 g/mol), their effect in polyols with higher molecular weight (≥1000 g/mol) and higher functionality (>2 hydroxyls per chain)—as are commonly used in polyurethane foams—has not been reported. Here, we show that the solubility of CO_2_ in polyether polyols decreases with molecular weight above 1000 g/mol and decreases with functionality using measurements performed by gravimetry-axisymmetric drop-shape analysis. The nonmonotonic effect of molecular weight on CO_2_ solubility results from the competition between effects that reduce CO_2_ solubility (lower mixing entropy) and effects that increase CO_2_ solubility (lower ratio of hydroxyl end groups to ether backbone groups). To generalize our measurements, we modeled the CO_2_ solubility using a perturbed chain-statistical associating fluid theory (PC-SAFT) model, which we validated by showing that a density functional theory model based on the PC-SAFT free energy accurately predicted the interfacial tension.

## 1 Introduction

Rigid polyurethane foams (RPUFs) are the leading, low-cost thermal insulation material available, outperformed only by relatively high-cost aerogels.^1^ RPUF’s exceptionally low thermal conductivity (≈20 mW/(m·K)^1^), ability to cure in place, 30-fold expansion to form tight seals, and low cost have made it the insulation of choice for refrigeration units, coolers, and even the fuel tanks for space shuttles.^2^ Unfortunately, its low thermal conductivity historically relied on volatile compounds like chlorofluorocarbons (CFCs) and hydrochlorofluorocarbons (HCFCs), which deplete the ozone.^3^ Hydrofluorocarbons (HFCs) and hydrocarbons (HCs) are currently used as nonozone-depleting alternatives to CFCs and HCFCs.^3^ Nevertheless, HFCs have a global warming potential over 1000 times greater than that of CO_2_ (1600 for HFC-134a vs 1 for CO_2_) and are targeted for phasing out by the Paris Climate Agreement (2015) and the Kigali amendment to the Montreal Protocol (2016).^4^ Additionally, HCs like isopentane and cyclopentane pose a high risk of flammability to consumers, even with the addition of flame retardants.^5^

In contrast, CO_2_ poses none of the hazards caused by the blowing agents listed above: it does not deplete the ozone, it is not flammable, and its use in foams has a low global warming potential.^3^ Relative to HFCs and HCs, however, CO_2_ has a higher thermal conductivity owing to its smaller molecular weight,^3^ so a CO_2_-blown insulating foam requires structural improvements like higher cell density and expansion ratio to have a competitive thermal conductivity. Both have been improved by increasing the solubility of CO_2_ in the polymer.^6−8^ Even in polyurethane foams, in which CO_2_ is typically produced *in situ* by chemical blowing, the equilibrium CO_2_ solubility can still affect foaming.^9^ Additionally, a high equilibrium CO_2_ solubility is desired in alternative polyurethane-foaming processes that predissolve CO_2_ in the polyol and isocyanate to achieve desired structures and minimize polyurea formation, which can compromise strength, stiffness, and processability.^10−12^

One approach to reach such high concentrations of dissolved CO_2_ is to prepare the CO_2_ in its supercritical state (above 7.39 MPa and 31.6 °C^13^). In thermoplastic foams, this approach has even yielded nanocellular foams (i.e., foams with a cell size <1 μm),^14^ which can achieve thermal conductivities comparable to those of aerogels because their small pore size slows gas conduction by the same “Knudsen effect”.^15^

Here, we focus on how the architecture of the polyol component of polyurethane affects the amount of CO_2_ that it can dissolve. We focus on the architecture of the polyol component instead of the isocyanate component of the polyurethane because CO_2_ is significantly more soluble in polyol than in isocyanate.^16^ Of the many aspects of polymer architecture, we consider the effects of molecular weight and functionality, i.e., the number of hydroxyl end groups per chain. Of the commonly used polyester and polyether polyols, we study polyether polyols owing to their wide range of hydroxyl functionality and molecular weight.^3^

The solubility of CO_2_ in polyether polyols has been shown to increase with higher pressure and lower temperature,^9,11^ but the effect of the architecture of the polyol has not been systematically studied in full. Parks and Beckman^17^ provided useful intuition for the effect of polyol architecture on CO_2_–polyol interactions in their study of the solubility of polyols in CO_2_.^17^ They noted that, because the carbon in CO_2_ has a lower electron density, it has a strong attraction to the relatively electron-rich ether groups along the polyol backbone, an affinity that has since been demonstrated with quantum mechanical calculations.^18^ In contrast, however, they reasoned that the CO_2_-phobicity of the hydroxyl groups, caused by their preference for self-interaction by hydrogen bonding,^18^ significantly decreases the solubility of short-chain polyols in CO_2_ owing to the high proportion of hydroxyl end groups to ether groups along the backbone.^17^ Solubility measurements indeed show that a hydroxyl group with a more CO_2_-philic end group like acetate^18^ or monomethyl ether^19^ increases mixing of polyol and CO_2_. Parks and Beckman^17^ concluded that the solubility of polyols in CO_2_—the opposite of the focus of the present work— increases with molecular weight for short chains but decreases with molecular weight for longer chains as the decrease in the entropy of mixing with molecular weight dominates the enthalpic gain of a higher ratio of ether to hydroxyl end groups.

In the context of CO_2_ solubility in polyols, Daneshvar et al.^20^ and Li et al.^21^ showed that the solubility of CO_2_ in poly(ethylene glycol) (PEG) increases with molecular weight for short chains (150–1000 g/mol), as did Yang et al.^11^ for various polyether polyols in the range of 255–1000 g/mol. Weidner et al.^22^ and Wiesmet et al.^23^ published measurements of CO_2_ solubility in longer PEG chains of 1500–8000 g/mol at higher temperatures between 50 and 100 °C but observed no statistically significant effect of molecular weight. On the basis of the work of Parks and Beckman^17^ and intuition from the Flory–Huggins model, we hypothesize that the solubility of CO_2_ decreases with molecular weight for long chains owing to the decreased entropy of mixing, with the trend tapering off at higher molecular weights and becoming more pronounced at lower temperatures. To our knowledge, however, this trend has not been demonstrated with experimental measurements in the literature.

The effect of functionality on CO_2_ solubility in polyether polyols has been investigated by Yang et al.,^11^ who reported a higher CO_2_ solubility in a 3-functional polyol (3 hydroxyl groups per chain) than in a 2-functional polyol of fixed molecular weight (1000 g/mol). This finding conflicts with the reasoning of Beckman and Parks that a higher concentration of hydroxyl groups should reduce the attraction of CO_2_ to polyol, so further study is in order. Uncovering these trends is important for selecting the polyol structure that optimizes CO_2_ solubility to achieve the desired thermal and mechanical properties in CO_2_-blown polyurethane foams.

In the present study, we systematically investigate the effect of molecular weight and functionality (number of hydroxyl groups per chain) on the solubility of CO_2_ in polyether polyols. We measure the solubility of CO_2_ using gravimetry-axisymetric drop-shape analysis (G-ADSA), which measures the change in mass of a sample upon absorption of CO_2_ using a magnetic suspension balance while simultaneously measuring the specific volume for precise accounting of the buoyancy force. Given the abundance of previous measurements of CO_2_ solubility in polyols with a molecular weight smaller than 1000 g/mol, we selected longer polyols of 1000 and 2700 g/mol. We hypothesized that, unlike the increase in CO_2_ solubility with molecular weight reported in the literature for shorter polyols (<1000 g/mol) whose interactions are strongly affected by their hydroxyl end groups, we would observe a decrease in CO_2_ solubility with molecular weight in these longer polyols (>1000 g/mol), consistent with the observations of Parks and Beckman^17^ for the solubility of polyols in CO_2_ and the decreased mixing entropy of longer polymer chains described in models like the Flory–Huggins. We also systematically varied the average functionality (number of hydroxyl groups per chain) from 2 to 4.7. Combined with those available in the literature, our measurements revealed a nonmonotonic dependence of the CO_2_ solubility on molecular weight, peaking around 1000 g/mol, and a monotonic decrease with hydroxyl groups per chain.

Because the G-ADSA measurement technique simultaneously measures the specific volume and solubility, we can fit parameters of a thermodynamic model to these measurements to estimate properties under conditions not measured. We model the system using the perturbed chain-statistical associating fluid theory (PC-SAFT)^24^ based on previous work^25^ and described in Section 4. This model can also provide the foundation for additional models of the interface, bubble growth, and bubble nucleation. Toward this end, we also briefly describe the use of a classical density functional theory (DFT) based on the free energy equation of PC-SAFT for modeling the interface between the polyol-rich and CO_2_-rich phases. We validate these predictions from DFT by comparison with the interfacial tension measured by G-ADSA. While the models accurately capture the CO_2_ solubility and interfacial tension, the present formulation of the PC-SAFT model underestimates the specific volume of the polyol-rich phase, likely because it does not account for associative interactions like hydrogen bonding, which become important in polyols shorter than 1000 g/mol.

## 2 Materials and Methods

**Figure 1:**
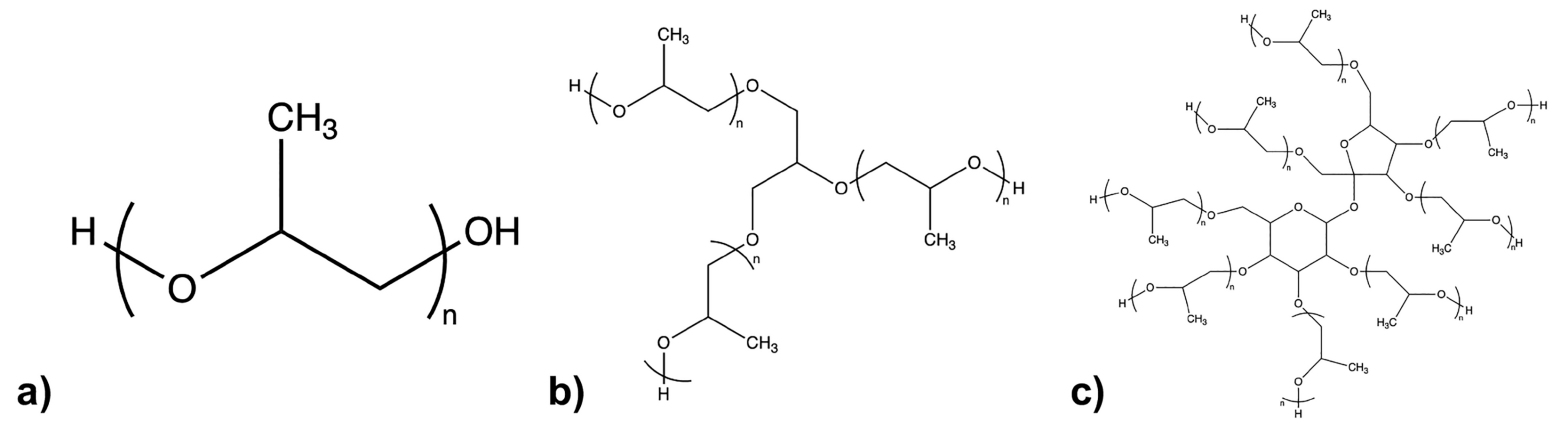
Molecular structure for (a) linear, (b) three-branched, and (c) eight-branched poly(propylene glycol) (PPG) polymers used in this study. The 1k2f polyol (see Table 1) is linear (a) with *n* = 17, the PPG polyol (2700 g/mol) is linear (a) with *n* = 46, the 1k3f polyol is three-branched (b) with *n* = 5, and the 1k5f polyol is a mixture of three-branched (b) with *n* ≈ 5 for each branch and eight-branched (c) with *n* between 1 and 2 for each branch.

We used gravimetry-axisymmetric drop-shape analysis (G-ADSA) for simultaneous measurements of CO_2_ solubility, specific volume, and interfacial tension in mixtures of polyol and CO_2_. With G-ADSA, we also simultaneously measured CO_2_ diffusivity, which is important for modeling the growth of bubbles that may nucleate from such mixtures, as explored in a recent thesis,^26^ but we do not discuss diffusivity in the present work. The G-ADSA technique combines weight measurements using a magnetic suspension balance (gravimetry) with pendant-drop analysis (axisymmetric drop-shape analysis).^27^ In the present work, we considered mixtures of CO_2_ and polyether polyols of various molecular weights and functionalities. The properties of these polyols are listed in Table 1 (proprietary chemicals are named by approximate molecular weight and hydroxyl functionality) in the range of 0–8 MPa at roughly 30 and 60 °C. Note that polydispersities were reported to be less than 1.1 by the commercial provider (Dow, Inc.). Additionally, we show the structures of the polyols in Figure 1.

**Table 1:** Properties of the Polyols Used in This Study^a^

name	*M*_*n*_ (g/mol)	*f*	ρ (g/mL)	η (mPa·s)	supplier
1k2f	1000	2	1.02	160	Dow, Inc.
1k3f	1000	3	1.02	290	Dow, Inc.
1k5f	728	4.7	1.084	4820	Dow, Inc.
PPG	2700	2	1.004	740	MilliporeSigma

a *M*_*n*_ = number-averaged molecular weight, *f* = functionality (number of hydroxyl groups per chain), ρ = density, and η = viscosity. Values reported are averages. Molecular weights, functionalities, and densities supplied by manufacturers. Viscosities were measured using an ARES shear rheometer (see Figure S3 in the Supporting Information). Density and viscosity measured at 25 °C and atmospheric pressure. Polydispersities are not known.

Here, we briefly describe the G-ADSA apparatus and technique. For a more thorough discussion, we refer the reader to the original publication of this method by Pastore Carbone et al.^27^

### 2.1 Apparatus

Briefly, a magnetic suspension balance (MSB, Rubotherm Prazisionsmesstechnik GmbH, Germany) held a Pyrex crucible with an inner diameter of 1.82 cm that contained the polyol sample. The crucible was suspended from the MSB by hooks with a volume of 2.267 35 mL, as measured in a helium atmosphere by Dr. Maria Rosaria Di Caprio of the Di Maio lab.^27^ A precise measurement of the volume of the hooks was necessary to estimate the buoyant force exerted on them by the CO_2_ atmosphere. A Teflon rod with a diameter of 2.05 mm was fitted snugly into a slot in the MSB to hold the pendant drop. The MSB was encased in a steel high-pressure cell, which was sealed around the MSB with a rubber O-ring. Two sapphire windows of a few centimeters in diameter machined in the cell provided a clear view of the pendant drop to a video camera with a convex objective lens.

### 2.2 Method

First, after an analytical balance was zeroed with the crucible, 1 mL of the desired polyol was poured into the crucible slowly enough not to entrain any bubbles. The weight of the sample under normal temperature and pressure was then measured with the analytical balance. Next, to prepare the pendant drop, a drop of polyol was first deposited onto the corner of a clean glass slide. The corner of the slide was then tilted over the upward-facing tip of the Teflon rod until a small drop (3–5 μL) dripped off and formed a hemisphere atop the tip of the rod. The rod was then inverted carefully to prevent loss of the drop and inserted into a slot in the MSB. The high-pressure steel encasement was then sealed around the MSB gently enough that the pendant drop would not fall. The high-pressure cell was enveloped in a second stainless steel jacket, which contained oil heated with a heating circulator (Julabo F25) to control the temperature of the sample. Once sealed, the pressure of CO_2_ inside the high-pressure cell was controlled using a Belsorp system. During the first stage of each experiment, moisture was removed from the polyol sample and the pendant drop by pulling a light vacuum (reduced pressure below 2 kPa) until the rate of decrease in the sample weight became extremely slow (less than 6 μg/min). If the sample weight did not stop decreasing within 20 min, we assumed that the additional mass loss resulted from the loss of the polyol, which could have short enough chains to be slightly volatile. The weight of the pure polyol sample and the volume of the pure polyol pendant drop were then measured.

The measurement of the pure polyol sample was followed by several measurements after pressurization with CO_2_. Pressurization was performed using the Belsorp system to slowly inject CO_2_ into the chamber. Above 5500 kPa, the Belsorp could not supply sufficient CO_2_ pressure to pressurize the chamber further, so we used an ISCO pump to pressurize the CO_2_ first before injecting it manually. Throughout each measurement, the MSB recorded the changing mass of the contents of the crucible as CO_2_ was absorbed into the polyol. The pressure was kept constant (within 20 kPa) until the mass did not change by more than 30 μg in 5 min. At this point, we considered the system to be sufficiently close to equilibrium for the error to be negligible. Upon reaching equilibrium, the MSB lowered the crucible until it rested on an overhanging platform, allowing the MSB to take three measurements of the tare weight of the MSB, which excluded the hooks, crucible, and contents of the crucible. At the same time, a video camera captured images of the pendant drop as it swelled from absorption of CO_2_, which were taken every few minutes. The Teflon rod swelled as well, as shown in Figure S1 of the Supporting Information. These measurements were repeated at ever higher pressures until the maximum pressure for the experiment was reached between 5 and 8 MPa. This maximum was determined to remain below the cloud point of polyol in the CO_2_-rich vapor phase, which was greater than 10 MPa for polyols of the molecular weights considered (see Parks and Beckman^17^). Next, we depressurized the system in steps by releasing CO_2_ through an automated ball valve from the high-pressure cell, taking measurements at each step. The release of CO_2_ was performed slowly enough that no nucleation of bubbles was observed in the pendant drop. Overall, we took measurements at 10–20 pressure values over the course of 1 week for each set of conditions. Note that the volume of the pendant droplet must be smaller to take measurements at higher pressures owing to the greater degree of swelling, which can cause the droplet to detach and fall if large enough (see discussion of the effect of droplet size in Di Caprio et al.^9^).

### 2.3 Computing Gas Solubility, Specific Volume, Gas Diffusivity, and Interfacial Tension from G-ADSA Measurements

The G-ADSA technique only directly measures an image of the drop shape, the total apparent weight of the crucible, attaching hooks, and sample, and the tare weight. The specific volume of the polyol–CO_2_ mixture, solubility of CO_2_ in the polyol, diffusivity of CO_2_ in the polyol, and interfacial tension between the polyol-rich and CO_2_-rich phases must therefore be calculated from these raw data. These calculations were performed with custom methods using open-souce Python packages, including jupyter,^28^ matplotlib,^29^ numpy,^30^ pandas,^31^ and scipy.^32^ The corresponding notebooks and libraries can be found in the GitHub repository andylitalo/G-ADSA.^33^

The general scheme of these calculations is summarized here. For further details, we refer the reader to the Supporting Information.

We first estimated the equilibrium volume of the pendant drop from its shape using the commercial software FTA32 (First Ten Angstroms). We then estimated the sample volume by assuming that its volume changes proportionally to the equilibrium volume of the drop. Next, we estimated the equilibrium sample mass using the MSB. The balance only directly measures the apparent weight and the tare weight. The difference between these measurements gives the sum of the masses of the sample, the crucible, and the supporting hooks minus the effect of the buoyancy force, which must be accounted for owing to the precision of these measurements. To compute the buoyancy force, we multiply the density of CO_2_ at the given pressure and temperature (available on the NIST Chemistry WebBook^13^) by the total volume of the weighed objects, which includes the volumes of the crucible, the supporting hooks, and the sample. The volume of the crucible and hooks was previously measured by Dr. Maria Rosaria Di Caprio in a helium atmosphere.^27^ After correction is made for buoyancy effects, the difference between the balance readings for the apparent weight and tare weight at zero pressure gives the mass of the dissolved gas. We then estimated the dry mass of the polyol by pulling a vacuum to remove dissolved vapor and moisture. The CO_2_ solubility is then the mass of dissolved gas divided by the total sample mass, equal to the sum of the mass of dissolved gas and the dry mass of the polyol. The specific volume of the sample can then be calculated by dividing the sample volume by the total sample mass.

Finally, we estimated the interfacial tension at a given pressure using axisymmetric drop-shape analysis (ADSA) performed with FTA32. This software automatically detects the edge of the drop and fits the contour predicted for a pendant drop predicted to its shape. When provided the density of the drop (reciprocal of the specific volume) and the density of the CO_2_-rich atmosphere (estimated using the *p*–*v*–*T* data for pure CO_2_ available from NIST^13^), the software computes the interfacial tension based on the pendant drop method.^34^ See the Supporting Information for further experimental details, analysis, and discussion of sources of error.

## 3 Results and Discussion

To explore the effect of polymer architecture on its capacity to dissolve CO_2_, we separately studied the effects of the functionality (hydroxyls per chain) and the molecular weight.

### 3.1 Effect of Functionality on CO_2_ Solubility

We begin by investigating how the solubility of CO_2_ in a polyol is affected by the polyol’s functionality, i.e., the average number of hydroxyls per chain. In this study, the number of hydroxyls per chain is also equal to the number of branches in the polymer backbone. When varying the functionality, we therefore vary both of these properties in conjunction, and we do not attempt to distinguish their individual effects in this study.

To our knowledge, measurements of the CO_2_ solubility in polyols with different functionality but fixed molecular weight have not been previously reported in the literature. While Yang et al.^11^ reported measurements of CO_2_ solubility in polyether polyols of different functionality, the molecular weights varied between chains. We considered the solubility of CO_2_ in a 2-functional (two hydroxyl groups per chain), 3-functional, and 4.7-functional (1k5f: polyol alkoxylated from a mixture of two initiators that yield a 3-functional and an 8-functional polyol). While the molecular weights of the polyols were not the same, they were similar, with average molecular weights of 1000, 1000, and 728 g/mol, respectively (see Table 1) and polydispersity indices below 1.1. We account for the effect of the slight difference in molecular weight in Section 3 of the Supporting Information to show that our conclusions are not affected by this difference.

**Figure 2:**
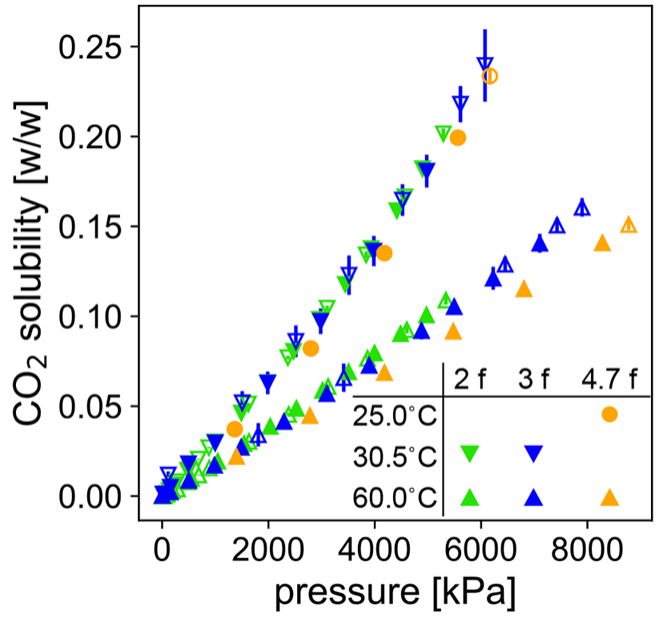
Solubility of CO_2_ in a polyol as a function of pressure (measured with G-ADSA) for polyols of three functionalities (average number of hydroxyl groups per chain): 2 (cyan), 3 (blue), and 4.7 (orange) (labeled as “ f” in the legend, where  is the functionality). Each polyol has an average molecular weight of 1000 g/mol, except that the 4.7f polyol (orange) has an average molecular weight of 728 g/mol. Polydispersities are below 1.1. Measurements were taken during both adsorption (filled markers) and desorption (open markers) of CO_2_ with agreement between the two within experimental uncertainty. Error bars may be smaller than glyphs of some data points. Data are shown in two temperature clusters: the upper cluster contains data measured at 30.5 °C for 2f and 3f polyols (downward triangles) and 25 °C for 4.7f polyol (circles); the lower cluster contains data measured at 60 °C (upward triangles).

To show the effect of polyol functionality on CO_2_ solubility, we plot the CO_2_ solubility for each of these polyols as a function of pressure at different temperatures in Figure 2. At low pressures, the difference in solubility is within the experimental uncertainty. At pressures above 3000 kPa, we can see that the polyol blend with the higher average number of hydroxyls per chain (4.7) has a significantly lower solubility of CO_2_ than the other polyols. At the higher temperature (60 °C, lower cluster of measurements) and above 4000 kPa, the polyol with the intermediate number of hydroxyls per chain (3) has a significantly lower solubility than the polyol with the fewest (2). This difference is less clear among the low-temperature data (upper cluster of measurements), in part because the CO_2_ solubility in the polyol blend with an average of 4.7 hydroxyls per chain was measured at a lower temperature (25 °C) than the others (30.5 °C) and because the 4.7-functional polyol has a lower molecular weight than the other (728 vs 1000 g/mol). Ideally, the measurements would have been taken at the same temperature and molecular weight as the other polyols, but we show in Figure S4 of the Supporting Information that accounting for these differences would yield a lower estimate of the CO_2_ solubility, further supporting our finding that the CO_2_ solubility decreases with functionality. This observation suggests that increasing the number of hydroxyls per chain decreases the solubility, with the difference becoming more apparent at higher pressures. This trend is consistent with the favorability of CO_2_–ether interactions over CO_2_–hydroxyl interactions reported in the literature.^17,18^

### 3.2 Effect of Molecular Weight on CO_2_ Solubility

**Figure 3:**
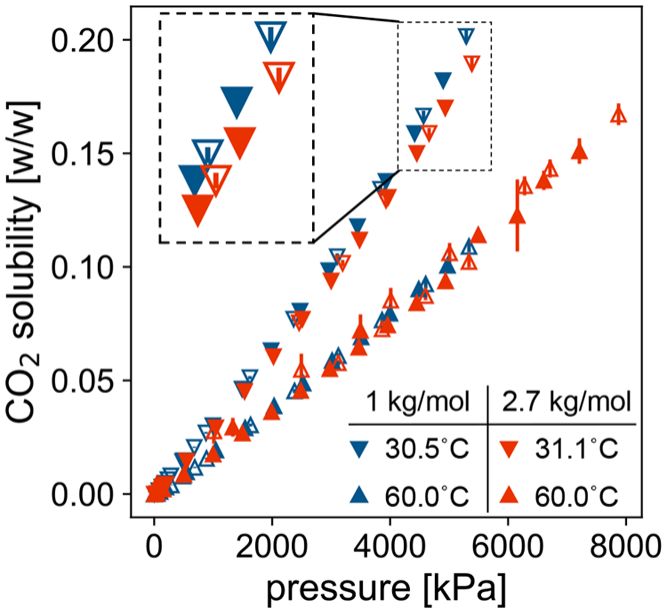
Solubility of CO_2_ in a polyol as a function of pressure (measured with G-ADSA) for 2-functional polyols with number-averaged molecular weights *M*_*n*_ of 1000 g/mol (blue) and 2700 g/mol (red). Measurements were taken during both adsorption (filled triangles) and desorption (open triangles) of CO_2_ in agreement within uncertainty. Error bars may be smaller than glyphs of some data points. Data are shown in two temperature clusters: the upper cluster contains data measured at 30.5 °C for 1000 g/mol (blue downward triangles) and 31.1 °C for 2700 g/mol (red downward triangles); the lower cluster contains data measured at 60 °C (upward triangles).

**Figure 4:**
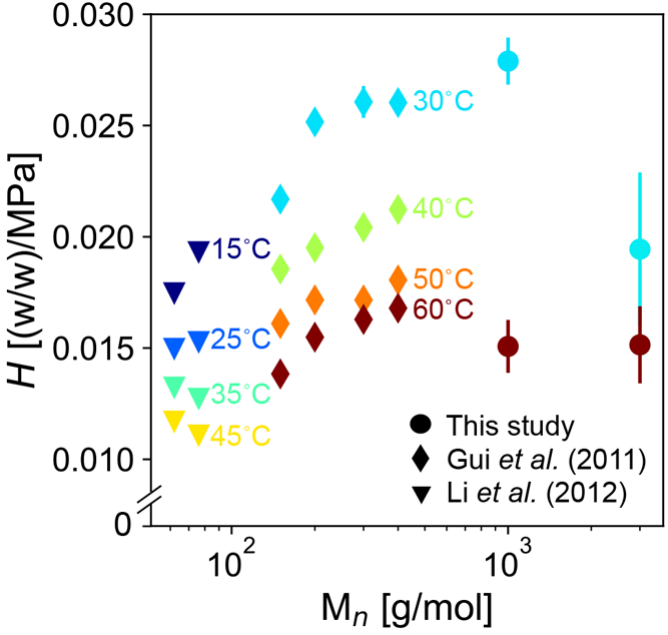
Henry’s constant for CO_2_ solubility in 2-functional polyols (two hydroxyls per chain) plotted as a function of the average molecular weight *M*_*n*_. Both literature data and the data measured with G-ADSA in the present study are provided. Error bars may be smaller than glyphs of some data points. Data are shown at different temperatures, which are indicated by the color and labeled in the plot. The symbols indicate the study from which the data came: this study (●), Gui et al. (⧫),^35^ and Li et al. (▼).^21^

Next, we considered the effect of molecular weight on CO_2_ solubility at a fixed functionality of 2. We used G-ADSA to measure CO_2_ solubility in two such polyols, one of 1000 g/mol and the other of 2700 g/mol, as shown in Figure 3. The solubility was slightly lower for the longer polyol at the lower temperature (30.5–31.1 °C, upper cluster of data). The solubility of the longer polyol was measured at a slightly higher temperature than that of the shorter polyol (31.1 °C for the longer vs 30.5 °C for the shorter), however, which could have also led to the lower measurement of solubility. The difference in solubility was not statistically significant at the higher temperature (60 °C, lower cluster of data). Because our measurements are not sufficient to robustly conclude that CO_2_ solubility decreases with molecular weight above 1000 g/mol, we turn to the literature to augment our data set. In particular, we combine our measurements with those of Gui et al.^35^ for the monomers ethylene glycol and propylene glycol and Li et al.^21^ for oligomers of poly(ethylene glycol) (PEG). While we measured CO_2_ solubility in poly(propylene glycol)-based polyols, which have an additional methyl group on each monomer when compared to PEG, we do not observe a systematic difference in the solubility of CO_2_ in PEG vs PPG, as seen in Figure 4 (compare the diamonds (PEG) to the circles (PPG)), so we do not investigate the effect of the additional methyl group in PPG.

To compare the CO_2_ solubility more clearly among measurements from the literature, we compared Henry’s constant, the rate at which the solubility increases with pressure at low pressures. We computed Henry’s constant by fitting the slope of a line passing through the origin to measurements of CO_2_ solubility at pressures below 1 MPa. We selected 1 MPa as the upper limit of data points to consider for estimating Henry’s constant based on our observation in Figure 2 that the solubility vs pressure increases superlinearly at higher pressures, deviating from Henry’s law. We report Henry’s constant in terms of weight fraction of CO_2_ per MPa [(w/w)/MPa] for comparison to other plots in this section. The only published measurements of polyether polyols that report solubility at pressures below 1 MPa were taken of 2-functional polyols (two hydroxyl OH groups per chain).^21,35^ Other measurements of CO_2_ solubility available in the literature either only report solubility at pressures well above 1 MPa^11,20,22,36−39^ or do not report a sufficiently precise experimental uncertainty for meaningful comparison to our measurements.^23,40^ Henry’s constant for the CO_2_ solubility in 2-functional polyether polyols is shown in Figure 4. We can see that Henry’s constant increases with molecular weight below ≈1000 g/mol, but it decreases with molecular weight above ≈1000 g/mol for a range of temperatures from 30 to 60 °C.

Although we considered the solubility of CO_2_ in a polyol-rich phase, it is interesting that Parks and Beckman^17^ measured that the solubility of a 2-functional polyether polyol in a CO_2_-rich phase was also highest in polyols with a molecular weight near 1000 g/mol, although they made measurements at a lower temperature (25 °C) and higher pressure (tens of MPa). While the solvation of polyol in CO_2_ is different than the solvation of CO_2_ in polyol, we suggest a similar explanation for the nonmonotonic dependence of the solvation of CO_2_ in polyols with molecular weight as that for the nonmonotonic dependence of the solvation of polyols in CO_2_ provided first by Parks and Beckman,^17^ as well as by others.^18,35,41^ As molecular weight increases, both the concentration of CO_2_-phobic hydroxyl groups (relative to ether linkages^18^) and the mixing entropy decrease. At low molecular weights, the decrease in the concentration of CO_2_-phobic hydroxyl groups is the dominant factor, which leads to greater CO_2_ solubility with molecular weight. At high molecular weights, the decrease in the mixing entropy is the dominant factor, which leads to lower CO_2_ solubility with molecular weight. We also note that we might expect the optimal molecular weight for CO_2_ solubility to decrease with temperature owing to the increased importance of entropy, which favors shorter chains. Our collection of data in Figure 4 appears to be consistent with this hypothesis at 60 °C, where the optimal molecular weight appears to be below 1000 g/mol, but further measurements are necessary to demonstrate this behavior robustly.

## 4 Modeling

Because the duration of a single set of G-ADSA measurements (one polymer, one temperature) can exceed a week, measuring the properties for all temperatures and pressures is not feasible. Instead, we developed a thermodynamic model to estimate some of the properties (solubility, specific volume) under conditions for which we lacked measurements. We chose to model the system with the perturbed chain-statistical associating fluid theory (PC-SAFT) equation^24^ on the basis of its success in modeling the solubility of CO_2_ in polystyrene and poly(methyl methacrylate).^25^ PC-SAFT also formed a suitable basis for the development of a classical density functional theory (DFT) for modeling the interfacial tension,^25^ which will be discussed later in this section. These can form the basis for an estimation of the energy barrier for bubble nucleation using the string method, which can be useful for models of foaming.^42,43^

The PC-SAFT equation of state provides a thermodynamic model for both pure components and mixtures. Unlike previous statistical associating fluid theory (SAFT) equations,^44,45^ where a hard-sphere system is used as a reference, PC-SAFT uses a hard-chain reference, from which a perturbation expansion is performed, leading to the prefix “perturbed chain”. This particular application of the perturbation theory combined with the fitting of some empirical parameters to measured properties of real polymers allows PC-SAFT to model the properties of polymer mixtures better than other SAFT equations (e.g., PR-SAFT^25^). Owing to our focus on experimental measurements, we provide only a brief, conceptual description of the PC-SAFT equation sufficient to introduce the parameters to be fitted; for a detailed mathematical description of the equation, see the work of Xu et al.^25^

The PC-SAFT equation is a mean-field theory with a free energy composed of three contributions: ideal, hard chain, and dispersion. The ideal contribution is the free energy of an ideal gas (noninteracting point particles). The hard-chain contribution is comprised of two terms. The first uses the Boublik–Mansoori–Carnahan–Starling–Leland (BMCSL) theory for mixtures of hard spheres with diameter σ_*i*_ for species *i*(46,47) to account for the excluded volume of hard spheres. The second term uses the perturbation theory (TPT1) developed by Wertheim^48,49^ and Chapman et al.^44^ to account for the excess free energy of association of *N*_*i*_ hard spheres of species *i* into a polymer chain through pairwise association. Finally, the dispersion contribution provides an empirical model of the interactions between pairs of molecules, in which these interactions are characterized by an energy parameter, ε_*ij*_, between two species *i* and *j*. This parameter is obtained from the energy parameter for a single species *i* by the following combining rule:
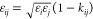
1where a temperature-dependent binary interaction parameter, *k*_*ij*_ = *AT* + *B* (where *T* is the temperature in Kelvin (K), *A* is in K^–1^, and *B* is dimensionless), is used to account for either favorable (*k*_*ij*_ < 0) or unfavorable (*k*_*ij*_ > 0) interactions between species *i* and *j*.

### 4.1 PC-SAFT Fits Solubility Measurements

Therefore, a complete PC-SAFT model of polyol and CO_2_ is described by eight parameters: two chain lengths *N*_CO2_ and *N*_polyol_, two bead diameters σ_CO2_ and σ_polyol_, two energy parameters ε_CO2_ and ε_polyol_, and the two parameters *A* and *B* defining the cross-interaction correction term *k*_CO2,polyol_ = *AT* + *B*. Thanks to previous work on polyol–CO_2_ mixtures by Xu et al.,^25^ the parameters specific to CO_2_ have already been fit to the pure-component equation of state data and have been validated against equation of state data from NIST.^13^ The parameters for the polyol and the cross interaction were fit by minimizing the root-mean-square error between the CO_2_ solubility measured by G-ADSA and that modeled by the present formulation of PC-SAFT. This parameter estimation was performed with the open-source package Clapeyron.jl^50^ (see Section 5 of the Supporting Information for details).

**Figure 5:**
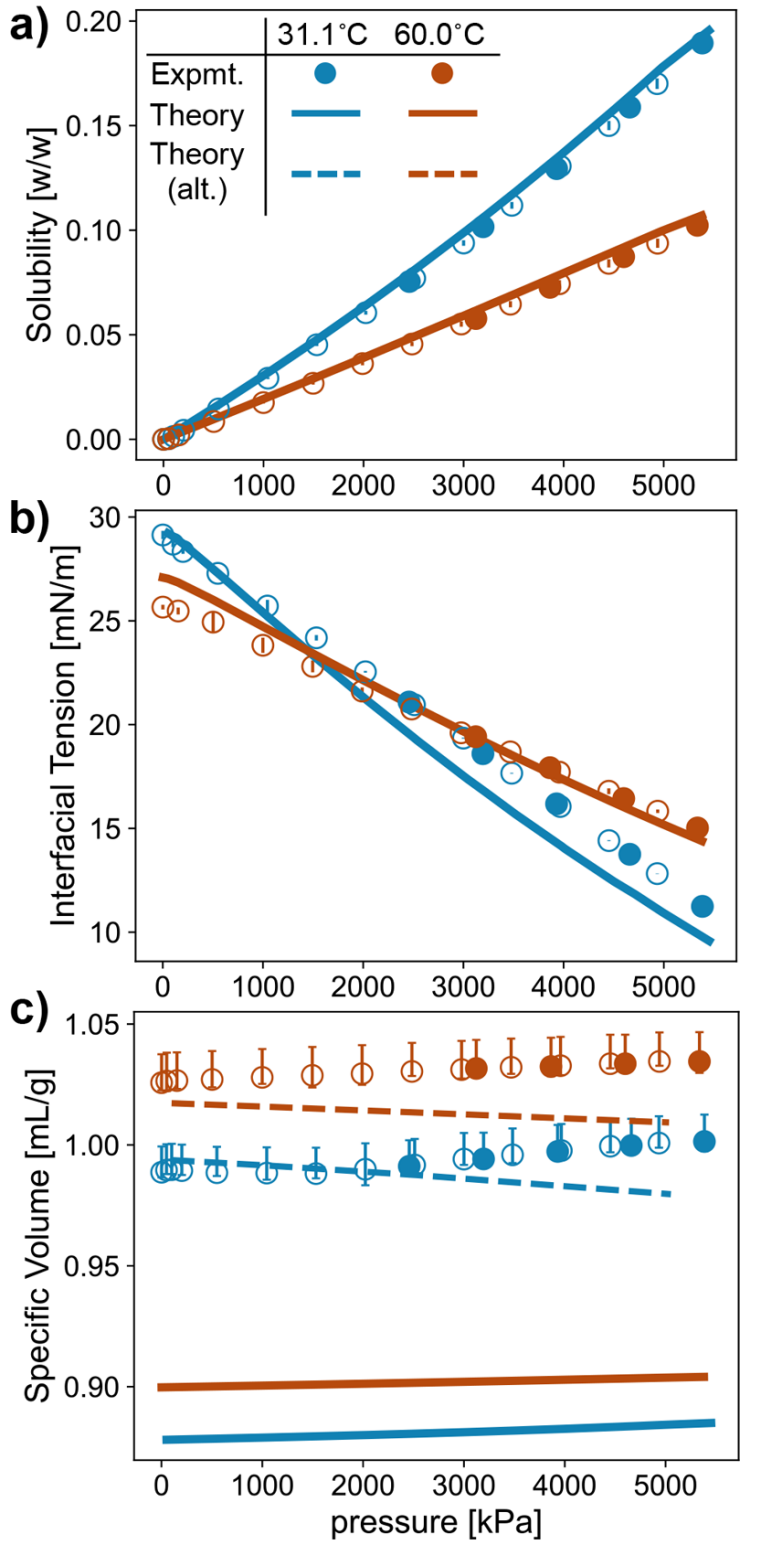
Comparison between experiment (circles) and theory (lines) for the (a) correlated solubility of CO_2_, (b) predicted interfacial tension, and (c) specific volume (solid lines represent predicted values, and dashed lines represent correlated values) of the polyol-rich phase as a function of pressure for PPG (2700 g/mol) at 31.1 °C (blue) and 60 °C (orange). Measurements were taken during CO_2_ adsorption (filled circles) and desorption (open circles). Error bars are shown but may be smaller than markers. In (c), the top error bar represents the systematic error of the experiment; the bottom error bar represents the statistical error of that particular measurement. Also shown in (c) is the correlated values of an alternative PC-SAFT model (dashed lines) for which σ_polyol_ = 3.17 Å and ϵ_polyol_ = 258 *k*_B_, as opposed to the values listed in Table 2 (explored further in Figure S8 of the Supporting Information).

As is often the case for PC-SAFT models of vapor–liquid equilibria, the present formulation of PC-SAFT can accurately model the CO_2_ solubility over a large range of degenerate parameters (see Figure S9 of the Supporting Information). To “break” this degeneracy, we selected parameters similar to those predicted using the group contribution method, a common method for estimating PC-SAFT parameters based on the functional groups present in the compounds.^51^ For PPG (2700 g/mol), the group contribution method predicted *N* = 122, σ = 3.23 Å, and ϵ = 233 *k*_B_, but these values did not produce an accurate model. Therefore, from this starting point, we minimized the root-mean-squared error between the model prediction and the measured CO_2_ solubility by using Clapeyron.jl.^50^ From this optimization, we obtained the PC-SAFT parameters for PPG (2700 g/mol) listed in Table 2. The estimates using these parameters are compared to the experimental measurements of solubility in Figure 5a.

**Table 2:** Parameters Fitted to the Solubility Data for PPG (2700 g/mol): *N*_*i*_ (Number of Beads per Chain), σ_*i*_ (Bead Diameter), and ε_*i*_ (Interaction Energy Parameter) for Species *i*^a^

species	*N*_*i*_ (beads)	σ_*i*_ (Å)	ε_*i*_ (*k*_B_)
CO_2_	2	2.79	170.5
PPG (2700 g/mol)	123	3.01	228.5

a ε_*i*_ is given in units of Boltzmann’s constant). The binary-interaction parameter between the two species is given as *k*_*ij*_ = 10^–4^(2.7*T* – 820), where *T* is the temperature in Kelvin. The corresponding model and experimental data are shown in Figure 5.

To demonstrate the precision of the model’s estimates, we show the sensitivity of our predictions of different properties to ±5% variations in the parameters σ_polyol_, ε_polyol_, and *N*_polyol_ for PPG in Figure S7 of the Supporting Information. This figure shows the sensitivity of the PC-SAFT predictions of CO_2_ solubility and specific volume and of the DFT predictions of interfacial tension, which are discussed below.

### 4.2 DFT Based on PC-SAFT Predicts Measured Interfacial Tension

On the basis of the PC-SAFT model described above, we developed a model of the interface between the polyol-rich and CO_2_-rich phases with classical density functional theory (DFT) following the method described in Xu et al.^25^ The result is a model that takes the PC-SAFT parameters fitted to experimental CO_2_ solubility data as input and predicts the equilibrium concentration profile of each species and the resulting interfacial tension between the two phases. An example of the equilibrium concentration profile of CO_2_ and PPG is shown in Figure S10 of the Supporting Information; here, we will focus on the predicted interfacial tension.

Because the parameters of the DFT model are determined purely from fitting the PC-SAFT model to the measured solubility and specific volume, we can test the validity of the DFT model by comparing its blind prediction to the measured interfacial tension, following the method described by Xu et al.^25^ The predicted interfacial tension between the CO_2_-rich and polyol-rich phases of a mixture of CO_2_ and PPG (2700 g/mol) is shown in Figure 5b. We see that the DFT model not only predicts the qualitative trends observed in the experimental measurements, such as decreasing interfacial tension with temperature at low pressure and increasing interfacial tension with temperature at high pressure (see Figure S6 in the Supporting Information for a discussion of this crossover), but also achieves reasonable, though not perfect, quantitative accuracy.

### 4.3 Present Formulation of PC-SAFT Cannot Accurately Model Specific Volume

Although the present formulation of the PC-SAFT equation accurately models CO_2_ solubility and the DFT based on it accurately predicts the interfacial tension for polyol–CO_2_ mixtures, the present formulation of PC-SAFT cannot accurately model the specific volume. While there is a large, degenerate set of PC-SAFT parameters for which the PC-SAFT model accurately estimates the measured CO_2_ solubility and the DFT model accurately predicts the measured interfacial tension, no group of parameters in that set yields an accurate PC-SAFT model of the specific volume (see Figure S9 of the Supporting Information). The disagreement between the specific volume measured with G-ADSA and the predictions of the PC-SAFT model based on the parameters in Table 2 is shown in Figure 5c.

Qualitatively, while the PC-SAFT model accurately captures the increase with temperature and the increase with pressure at high pressures of the specific volume, it fails to capture the nonmonotonic dependence of the specific volume on pressure at low pressures and temperatures. The Di Maio group has previously reported a similar nonmonotonic dependence of the specific volume on pressure from measurements with G-ADSA for a formulation of polyether polyols^9^ and for poly(caprolactone) (PCL).^52^ They further demonstrated that this behavior is the result of different packing densities of CO_2_ in the polymer matrix at different pressures using evidence from Raman spectroscopy.^53−55^ This failure of PC-SAFT’s coarse-grained beads to capture the nonmonotonic effect of pressure on specific volume is not surprising given that it arises owing to finer molecular structures.

Quantitatively, however, the present formulation of PC-SAFT with the parameters in Table 2 underestimates the specific volume (overestimates the density) by over 10%. While increasing the parameters σ_polyol_ and ϵ_polyol_ can improve the quantitative agreement between theory and experiment for the specific volume without affecting the estimates of the solubility and interfacial tension (see Figure S8 in the Supporting Information), it leads to a model for which the specific volume decreases with pressure; however, the experimental measurements of this work and in the literature show that the specific volume generally increases with pressure. Given the non-negligible association between CO_2_ and the hydroxyl groups of the polyol, as well as among the hydroxyl groups, a more accurate PC-SAFT model of the specific volume would account for these association interactions. Additional experimental measurements of the specific volume of pure polyol at different pressures would also improve the fitting of the polyol-specific PC-SAFT parameters. Nevertheless, while our PC-SAFT model fails to estimate the specific volume accurately, we present it here given its accuracy in fitting the CO_2_ solubility and predicting the interfacial tension.

## 5 Conclusions

We have performed high-precision measurements of the solubility of CO_2_ in polyether polyols with a molecular weight over 1000 g/mol using the G-ADSA technique.^27^ Combined with measurements of the solubility of CO_2_ in other polyols available in the literature, these results revealed that the solubility increases with molecular weight below 1000 g/mol and decreases with molecular weight above 1000 g/mol. This nonmonotonic effect of molecular weight on CO_2_ solubility in polyether polyols is consistent with the trends in the cloud point of polyether polyols dissolved in CO_2_ reported by Parks and Beckman.^17^ We have also performed systematic measurements of CO_2_ solubility in polyols with different numbers of hydroxyl end groups per chain. We showed that increasing the number of hydroxyl groups per chain decreases CO_2_ solubility, which is consistent with previous observations that hydroxyl end groups have less-favorable interactions with CO_2_ than the ether linkages along the backbone.^17,18^

Furthermore, we showed that a perturbed chain-statistical associating fluid theory (PC-SAFT) model can accurately describe the solvation of CO_2_ in polyether polyols. We extended the model based on the method presented by Xu et al.^42^ to a density functional theory (DFT), which accurately predicted the interfacial tension as well. While the present formulation of PC-SAFT is unable to estimate the specific volume of the polyol-rich phase accurately, we are hopeful that the proper incorporation of the association interactions among the polyol’s hydroxyl end groups and additional equation-of-state data can yield a more accurate model.

## Supporting Information Available

G-ADSA apparatus, data analysis, measurement uncertainties, and reproducibility; viscosity measurements of polyols; effects of dissolved CO_2_ fraction and temperature on interfacial tension; sensitivity of PC-SAFT and DFT to variations in parameters; DFT prediction of nonmonotonic CO_2_ concentration profile (PDF)
