# Supplementary material for: Competition between CO2-philicity and Mixing Entropy Leads to CO2 Solubility Maximum in Polyether Polyols
Source: Ind Eng Chem Res. 2022 Aug 18;61(34):12835–44. doi: 10.1021/acs.iecr.2c02396 (PMC9438450; doi:10.1021/acs.iecr.2c02396)
Supplement: Supplementary file 1 — ie2c02396_si_001.pdf [file ie2c02396_si_001.pdf]

Supporting Information: Competition between CO2-philicity and mixing entropy leads to CO2 solubility maximum in polyether polyols

Andrew S. Ylitalo,† Huikuan Chao,‡ Pierre J. Walker,† Jacob Crosthwaite,‡ Thomas C. Fitzgibbons,¶ Valeriy G. Ginzburg,§ Weijun Zhou,¶ Zhen-Gang Wang,† Ernesto Di Maio,‖ and Julia A. Kornfield*,†

†*Division of Chemistry and Chemical Engineering, California Institute of Technology, Pasadena, California 91125, United States*

‡*Dow, Inc., Midland, MI 48667, United States*

¶*Dow, Inc., Lake Jackson, TX 77566, United States*

§*Michigan State University, East Lansing, MI 48910, United States*

‖*Dipartimento di Ingegneria Chimica, dei Materiali e della Produzione Industriale, University of Naples, Federico II, P. le Tecchio 80, 80125 Naples, Italy*

E-mail: jak@cheme.caltech.edu

Phone: +1 626-395-4138

Contents

List of Figures **S-2**

1 Gravimetry–Axisymmetric Drop Shape Analysis (G-ADSA) **S-4**

1.1 Apparatus S-4

1.2 Data Analysis S-4

1.3 Propagating Uncertainty in G-ADSA Measurements S-8

1.4 Reproducibility of Results S-9

2 Measurement of Viscosity of Polyols **S-10**

3 Estimate Effects of Temperature and Molecular Weight on CO_2 Solubility in 4.7-functional Polyol **S-10**

4 Distinguishing Effects of Dissolved CO_2 Fraction and Temperature on Interfacial Tension **S-15**

5 Sensitivity of PC-SAFT and DFT Models to Variations in Parameters **S-16**

5.1 Alternative Parameters S-19

6 DFT Predicts Non-monotonic CO_2 Concentration Profile **S-24**

References **S-25**

List of Figures

S1 Swelling of Teflon rod from CO_2 absorption S-5

S2 Comparison of repeated measurements under identical conditions show reproducibility of G-ADSA measurements S-11

S3 Shear rheometry of polyols S-12

S4 Model effects of temperature and molecular weight on CO_2 solubility in 4.7-functional polyol S-13
S5 Method of estimating effect of molecular weight on CO_2 solubility in 4.7-functional polyol S-14
S6 Interfacial tension of polyol–CO_2 mixture vs. pressure, solubility S-16
S7 Sensitivity of PC-SAFT model predictions to variations in its parameters . . S-18
S8 Plots comparing PC-SAFT model predictions of measured thermophysical parameters with two sets of parameters S-20
S9 Heat maps of different objective functions for mixture of polyol and CO_2 for different values of ϵ, $N_{2.7\text{k}}$ and σ S-22
S10 Density profile of polyol (orange) and CO_2 (blue) at interface between liquid and vapor phases predicted by DFT S-24

1 Gravimetry–Axisymmetric Drop Shape Analysis (G-ADSA)

1.1 Apparatus

As described in the main text, the Teflon rod swelled as a result of absorbing CO_2 as the pressure increased. This swelling is depicted in Figure S1. An image of the interior of the G-ADSA apparatus is shown in panel (a) as well for reference, with the white Teflon rod (about 3 cm in length) visible above the hanging crucible. The tip of the Teflon rod is outlined in a black box and is shown with a pendant droplet of polyol in panels (b) and (c). The elongation due to swelling from CO_2 can be seen in the apparent descent of the tip of the rod from panel (b) (no CO_2 atmosphere) to panel (c) (high-pressure CO_2 atmosphere). Note that the Teflon rod elongates by less than 1 mm due to swelling from CO_2, amounting to a few percent of the overall length.

1.2 Data Analysis

Having briefly described the methods for estimating CO_2 solubility, specific volume, interfacial tension, and CO_2 diffusivity for polyol–CO_2 mixtures using G-ADSA measurements, we now describe the analysis in greater depth and mathematical detail below. This analysis is encoded in the GitHub repository `andylitalo/g-adsa`.[S1]

We begin by estimating the equilibrium volume of the pendant drop from the image of the drop shape using the commercial software FTA32 developed by First Ten Angstroms. The software only requires that we click the leftmost and rightmost points of contact between the pendant drop and the Teflon rod and provide a length scale for the pixels in the image. To determine the length scale, we divide the known width of the tip of the rod (measured with calipers) by its width in pixels in the image taken under vacuum—at higher pressures, the Teflon rod swells. Finally, the software estimates the equilibrium drop volume V_{drop} by

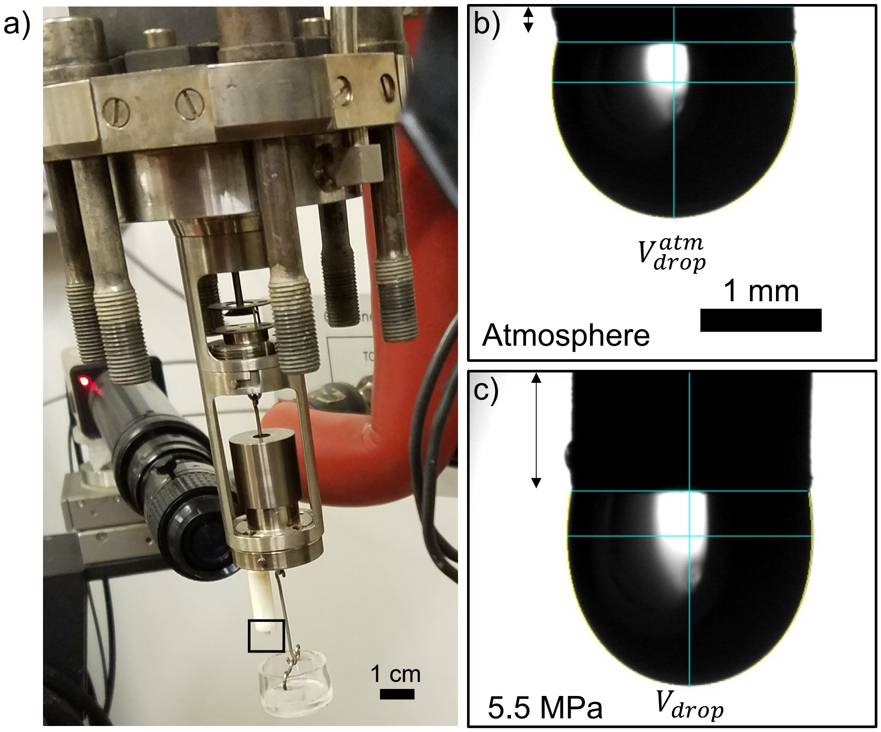

Figure S1: Swelling of Teflon rod suspending pendant drop in G-ADSA due to absorption of CO_2. a) Interior of G-ADSA setup. The top of the white Teflon rod is fitted into the bottom of the stainless steel housing of the magnetic suspension balance. The tip of the rod is outlined in a black square. The tip as viewed by the camera seen behind the rod is shown in panels (b) and (c). b) Close-up image of the tip of the Teflon rod and pendant drop under atmospheric pressure at 30.5 °C. c) Close-up image of the tip of the Teflon rod and pendant drop under 5.5 MPa of CO_2 at 30.5 °C. Note that the tip of the Teflon rod has descended about 1 mm due to the swelling from absorption of CO_2.

assuming axisymmetry. We assume that the equilibrium drop volume $V_{drop}(p)$ grows with pressure p in proportion to the equilibrium sample volume $V_{samp}(p)$,

$$V_{samp}(p) = \frac{V_{drop}(p)}{V_{drop}(0)} V_{samp}(0) \tag{1}$$

Next, we estimate the equilibrium sample mass. In this Section, we will use the letter B to indicate a direct measurement of force by the Rubotherm balance in the G-ADSA apparatus (scaled by the gravitational acceleration g to be in units of mass), m to indicate mass, $\tilde{m}$ to indicate a force on the balance (buoyancy) scaled to be in units of mass, and w to indicate a mass fraction (kg/kg). The balance provides us readings of the apparent weight

$B_{app}(p)$ and the tare weight $B_{tare}(p)$ at the given pressure. Because these weights are scaled by the gravitational acceleration, they correspond to the mass of the components of the scale minus the buoyancy force from the surrounding atmosphere. Thus, the difference between these measurements $\Delta B(p) \equiv B_{app}(p) - B_{tare}(p)$ is equivalent to the sum of the mass of the sample at the equilibrium pressure $m_{samp}(p)$, the mass of the crucible m_{cruc}, and the mass of the hooks m_{hook} minus the buoyancy force scaled by gravitational acceleration $\tilde{m}_{buoy}(p)$ at the equilibrium pressure (note that the buoyancy force must be accounted for due to the precision of these measurements),

$$\Delta B(p) = m_{samp}(p) + m_{cruc} + m_{hook} - \tilde{m}_{buoy}(p) \tag{2}$$

When the pressure $p = 0$, the mass of the sample is the dry mass of the polyol $m_{samp}(p = 0) \equiv m_{poly}$ (assumed to be constant, *i.e.*, negligible vaporization of polyol) and there is no buoyancy force ($\tilde{m}_{buoy}(p = 0) = 0$). Thus, the difference in balance readings at zero pressure is

$$\Delta B(p = 0) = m_{poly} + m_{cruc} + m_{hook} \tag{3}$$

Subtracting equation 3 from equation 2 gives

$$\Delta B(p) - \Delta B(p = 0) = m_{samp} - m_{poly} - m_{buoy}(p)$$

The sample mass m_{samp} is composed of the mass of the dry polyol m_{poly} and the mass of the gas m_{gas}, such that the equation above can be solved for the mass of the gas,

$$m_{gas}(p) = \Delta B(p) - \Delta B(p = 0) + m_{buoy}(p) \tag{4}$$

We now only require the effect of the buoyancy force on the balance reading $\tilde{m}_{buoy}(p)$ to compute the mass of dissolved gas. When scaled by gravitational acceleration, the buoyancy force on the balance reading is the density of the CO_2 atmosphere at the given pressure and temperature T, $\rho_{CO2}(p, T)$ (available on the NIST Chemistry WebBook[S2]), multiplied by the total volume of weighed objects V_{tot}. The total volume of weighed objects includes the volumes of the crucible V_{cruc}, the supporting hooks V_{hook}, and the sample $V_{samp}(p)$. Thus, the buoyancy force is

$$\tilde{m}_{buoy}(p) = \rho_{CO2}(p)[V_{cruc} + V_{hook} + V_{samp}(p)] \tag{5}$$

The volume of the crucible and hooks $V_{cruc} + V_{hook}$ was measured to be 2.2675 mL by Dr. Maria Rosaria Di Caprio in a helium atmosphere before performing experiments, as described in previous work.[S3] The volume of the sample at the given pressure $V_{samp}(p)$ can be calculated with equation 1.

The mass of dissolved gas can thus be computed by plugging the result of equation 5 into equation 4. To compute the solubility of CO_2 in the polyol, we also need the dry mass polyol, m_{poly}. While we measured the polyol mass in the atmosphere beforehand, we expect that the polyol had absorbed some of the moisture from the humid, Neapolitan summertime atmosphere. We therefore solve equation 3 for m_{poly}

$$m_{poly} = \Delta B(p = 0) - (m_{cruc} + m_{hook}) \tag{6}$$

and plug in the known difference in balance readings at zero pressure $\Delta B(p = 0)$ and mass of the crucible and supporting hooks $m_{cruc} + m_{hook}$ to compute the dry polyol mass. Having computed the gas mass at the given pressure $m_{gas}(p)$ and dry polyol mass m_{poly}, we compute the solubility by mass of CO_2 in the polyol,

$$w_{CO2}(p) = \frac{m_{gas}(p)}{m_{gas}(p) + m_{poly}} \tag{7}$$

Next, we compute the specific volume of the sample, which is the sample volume $V_{samp}(p)$ divided by the sample mass $m_{samp}(p)$. Noting that the sum of the gas mass and dry polyol mass gives the sample mass $m_{samp}(p) = m_{gas}(p) + m_{poly}$, the specific volume is

$$v(p) = \frac{V_{samp}(p)}{m_{samp}(p)} \tag{8}$$

Finally, we estimate the interfacial tension at a given pressure $\gamma(p)$ using axisymmetric drop shape analysis (ADSA) performed with the commercial software First Ten Angstroms 32 (FTA32). This software automatically detects the edge of the drop and fits the contour predicted for a pendant drop predicted to its shape. When provided the density of the drop (reciprocal of the specific volume $1/v(p)$) and the density of the CO_2-rich atmosphere (estimated using the pvT data for pure CO_2 available from NIST[S2]), the software computes the interfacial tension. To learn more about the pendant drop method and its estimation of interfacial tension, see the work of Song and Springer.[S4] Because ADSA was performed using a camera separate from the Rubotherm MSB used for gravimetry, the two sets of measurements were synchronized to a common start time, thereby providing simultaneous measurements of each parameter.

The diffusivity can also be computed from the transient measurements of dissolved CO_2 while the sample reaches equilibrium. Because we focused on equilibrium properties, we do not discuss the calculation of the diffusivity here. Those who are interested may refer to the original publication of the G-ADSA method[S3] as well as detailed derivations provided in a related dissertation.[S5]

1.3 Propagating Uncertainty in G-ADSA Measurements

To estimate the error bars shown in the plots of G-ADSA measurements, we propagated the uncertainty from all known sources of error through the analysis equations. These formulas are encoded in the `errprop.py` library of functions within the `andylitalo/g-adsa`

GitHub repository.[S1]

1.4 Reproducibility of Results

In addition to precise, measurements with G-ADSA are reproducible. We demonstrate their reproducibility in two ways. First, we showed in the main text that the measurements made while pressurizing the system are generally statistically indistinguishable from those made while depressurizing the system—which may take place days later—with few exceptions. This agreement shows that there is not significant loss of polyol due to solvation in the CO_2-rich vapor phase, which would otherwise compound over the course of the experiment and lead to an increasing discrepancy between the measurements of solubility and specific volume made during pressurization and depressurization. While we cannot rule out the loss of some polyol to solvation in the CO_2-rich phase (although, based on PC-SAFT estimates, the loss of polyol is less than a μg out of a 1 g sample), our observation that measurements during pressurization and depressurization agree within experimental uncertainty indicates that the effect of the loss of polyol on our measurements is smaller than the effect of other sources of error. Second, we show that measurements from two separate experiments run under the same conditions are also generally statistically indistinguishable. We demonstrate this reproducibility using PPG (2700 g/mol) at 60 °C in Figure S2 for the solubility of CO_2 (a), interfacial tension (b), and specific volume of the polyol-rich phase (c). For each property, the measurements made during pressurization (filled symbols) are statistically indistinguishable from those made during depressurization (open symbols), indicating that the measurements were taken near equilibrium, as desired. The one exception is the measurement of the interfacial tension at low pressures in trial 2, which was significantly lower during depressurization than during pressurization. The cause is most likely residual CO_2 because we depressurized the system in larger steps than those with which we pressurized the system, and the CO_2 solubility is slightly higher as well. Therefore, reaching equilibrium would take longer, but due to limited time, we could not always wait to reach equilibrium. The

measurements from the two experiments (blue and magenta) are generally indistinguishable, especially those of the specific volume (c). The primary exception is that the CO_2 solubility was measured to be slightly higher in trial 2 than in trial 1 at pressures above 2000 kPa, which may have resulted in slight variations in the preparation of the sample that were not accounted for in the uncertainty analysis.

As a further test of the reproducibility of these measurements, we compared them to data from the literature. Due to the length of that discussion and the focus of the present work on measurements made at lower pressures, we refer the reader to the related thesis for details.[S5]

2 Measurement of Viscosity of Polyols

The viscosities of the polyols used in this study that were reported in Table 1 were measured using an ARES RFS shear rheometer with 50 mm parallel plates, as shown in Figure S3. Measurements of shear stress were taken over a shear rate sweep from 1 1/s to 100 1/s at a temperature of 25 °C. The viscosity remains roughly constant across the shear rate sweep, indicating Newtonian behavior.

3 Estimate Effects of Temperature and Molecular Weight on CO_2 Solubility in 4.7-functional Polyol

In Figure 1, the solubility of CO_2 in three polyols of similar molecular weight and different functionality is compared. While the CO_2 solubility appears to decrease with increased functionality (from 2f to 3f to 4.7f), the parameters of the measurement for the 4.7-functional polyol are not exactly the same, so a precise comparison cannot be made. Specifically, the 4.7-functional polyol is 25 % shorter than the others, having an average molecular weight of 728 g/mol rather than 1000 g/mol according to the manufacturer (Dow, Inc.). Additionally,

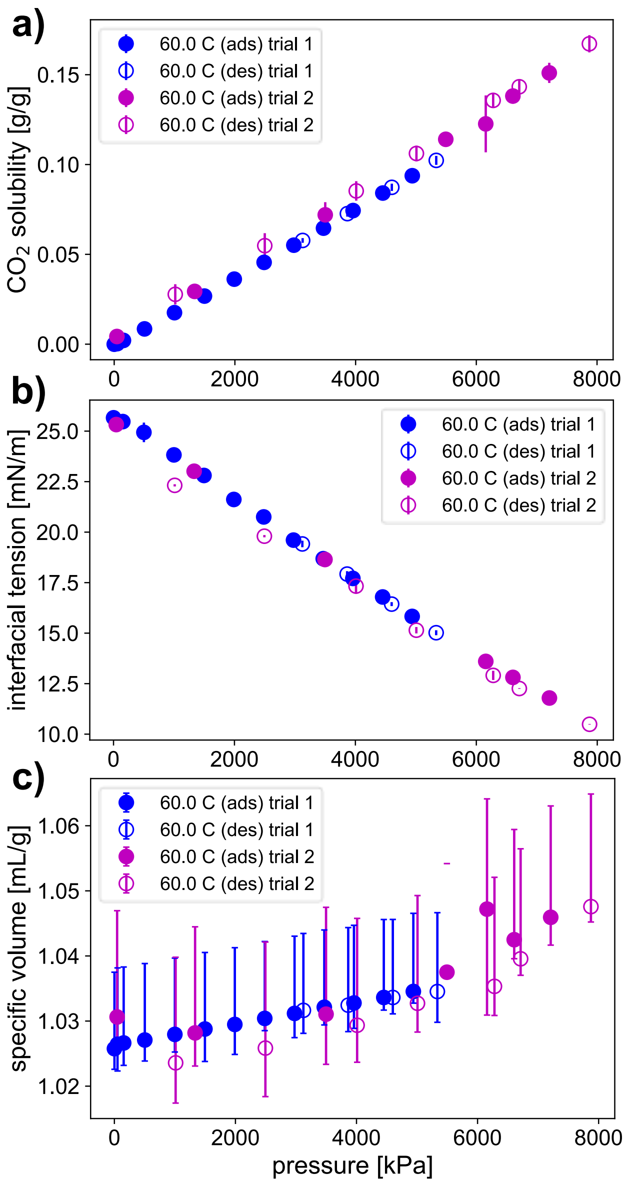

Figure S2: Measurements from two experiments of (a) the solubility of CO_2, (b) the interfacial tension, and (c) the specific volume of the polyol-rich phase for a mixture of CO_2 and PPG (2700 g/mol) at 60 °C. Measurements taken during pressurization (adsorption of CO_2) are shown with filled circles while those taken during depressurization (desorption of CO_2) are shown with open circle. The measurements taken from the first trial of the experiment are shown in blue and those taken from the second trial are shown in magenta. Error bars are shown, though they may be smaller than the symbol. In (c), the top error bar represents the systematic error of the experiment while the bottom error bar represents the statistical error of that particular measurement. Measurements are generally statistically indistinguishable, except for the solubility at high pressures and the interfacial tension at lower pressures between experiments.

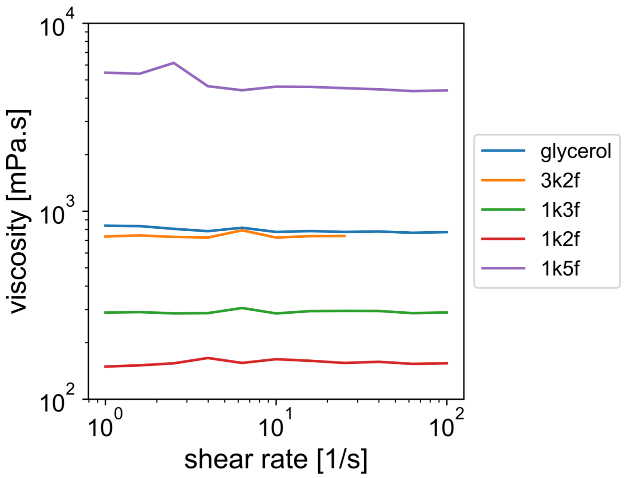

Figure S3: Viscosity measured in a shear rate sweep from 1 1/s to 100 1/s at a temperature of 25 degrees Celsius with 50 mm parallel plates and 0.7 mm gap size on an ARES RFS shear rheometer. Polyol names are encoded to conceal the identities of proprietary chemicals; for properties, see Table 1 in the main text.

the low-temperature measurement of the 4.7-functional polyol was performed at a lower temperature (25 °C) than was that of the other polyols (30.5 °C).

To distinguish the effect of functionality more clearly, we estimated the effects of molecular weight and temperature in this measurement, as shown in Figure S4. The effect of temperature can be estimated using a PC-SAFT model, which was developed to model the branched structure of the 4.7-functional polyol. In this branched model, a new species is added to represent the hydroxyl end groups. These are given a high value of the ϵ parameter ($_{OH} = 265\ k_B$) than the backbone $\epsilon_{bb} = 259\ k_B$) to model the greater affinity of CO_2 to the backbone than the end groups noted in literature.[S6] After fitting the model to the measurements of CO_2 solubility for the 4.7-functional polyol shown in Figure 1 of the main text, we used the model to estimate the solubility at 30.5 °C (yellow dashed line in Figure S4), which was lower than the solubility measured at 25 °C (yellow circles in Figure S4).

To estimate the effect of increasing the molecular weight of the 4.7-functional polyol from the true average value of 728 g/mol to the average value of the 2-functional and 3-functional polyols of 1000 g/mol, we could not use the PC-SAFT model because it does not account for the association of the hydroxyl end groups that leads to the non-monotonic dependence

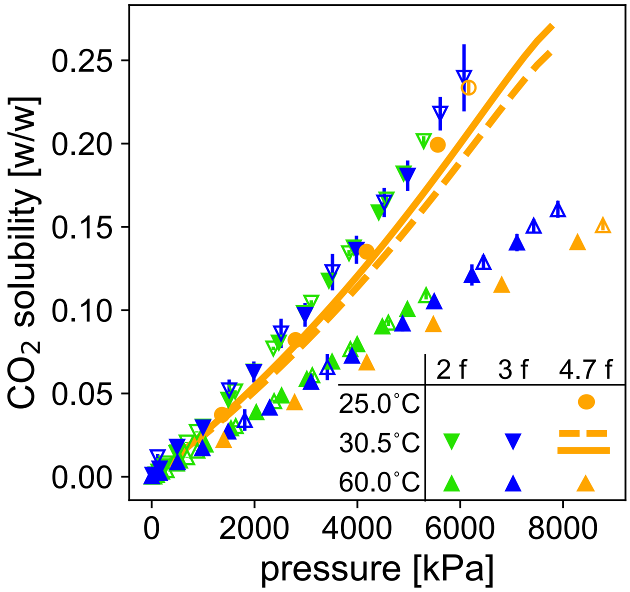

Figure S4: Reproduction of Figure 1 from the main text showing the effect of functionality on the solubility of CO_2 in polyol, here with two estimates of the solubility in a 4.7-functional polyol. The first estimate uses a PC-SAFT model to estimate the effect of increasing the temperature from 25 °C (measurement shown in yellow circles) to 30.5 °C to match the temperature of the low-temperature measurements for the 2-functional and 3-functional polyols (yellow dashed line). The second estimate uses the effect of molecular weight on Henry's constant to estimate the effect of increasing the molecular weight from 728 g/mol (true value) to 1000 g/mol (value for the 2-functional and 3-functional polyols) on Henry's constant at 30 °C, as shown in Figure S5, and assumes that the CO_2 solubility would increase by the same fraction (solid yellow line).

of CO_2 solubility on the molecular weight. Instead, we estimated the effect by extrapolating from the measured increase in Henry's constant based on the data for 2-functional polyols in Figure 3 of the main text, which is reproduced for convenience in Figure S5. In the Figure, Henry's constant increases from 0.0260 ± 0.0005 (w/w)/MPa at 400 g/mol to 0.028 ± 0.001 (w/w)/MPa at 1000 g/mol. While a linear interpolation may not be accurate due to the non-monotonicity, the downward concavity of the trendline means that any non-monotonicity would only reduce the increase in Henry's constant with molecular weight from 728 g/mol to 1000 g/mol because Henry's constant at 728 g/mol would be higher.

Therefore, to estimate the largest increase in Henry's constant with molecular weight consistent with the reported measurements, we use a linear interpolation from the lower

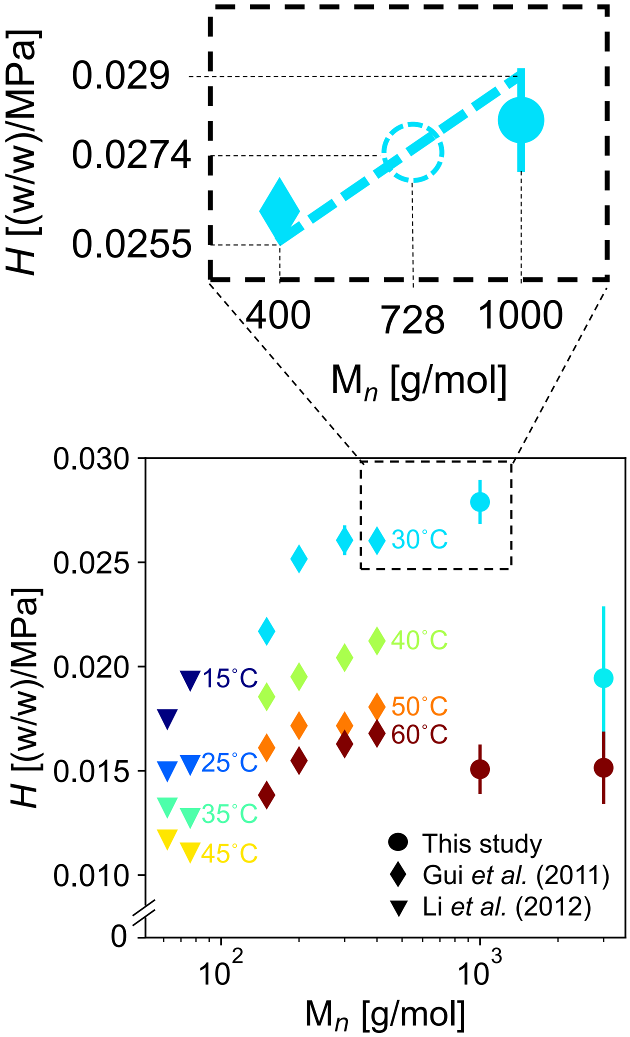

Figure S5: Figure 3 from the main text is reproduced at the bottom, showing Henry's constant H in weight fraction of dissolved CO_2 per MPa as a function of number-averaged molecular weight M_n [g/mol]. At the top, the section of the plot used to estimate the effect of molecular weight on Henry's constant at 30 °C between 400 g/mol and 1000 g/mol is shown. As an upper bound on the estimate, the greatest slope within the experimental uncertainty is assumed (dashed blue line). The estimated Henry's constant at 728 g/mol, the molecular weight of the 1k5f polyol, is shown (blue dashed circle). The fractional increase in Henry's constant from 728 g/mol to 1000 g/mol for these 2-functional polyols is used as a rough estimate of the increase in the CO_2 solubility in the 1k5f polyol if its molecular weight were increased from its true value of 728 g/mol to 1000 g/mol. The coordinates of each point on the interpolation line are indicated by thin dashed gray lines.

end of the uncertainty in the measurement at 400 g/mol (0.0255 (w/w)/MPa) to the upper end of the uncertainty in the measurement at 1000 g/mol (0.029 (w/w)/MPa) to estimate a Henry's constant of 0.0274 (w/w)/MPa at 728 g/mol. This linear interpolation is shown

in the zoomed-in section of the plot shown in the top of Figure S5. The increase in Henry's constant from 728 g/mol to 1000 g/mol would then at most be 0.0274 (w/w)/MPa to 0.029 (w/w)/MPa, which is an increase of 5.8 %. In Figure S4, we increased the solubility of CO_2 estimated by the PC-SAFT model at 30.5 °C (yellow dashed line) by 5.8 % as an estimate of the solubility of CO_2 in a hypothetical 4.7-functional polyol of molecular weight 1000 g/mol at 30.5 °C. These are the identical temperature and molecular weight of the low-temperature measurements of CO_2 solubility in the 2-functional and 3-functional polyols. Even after accounting for the effects of the discrepancy in these parameters in the original measurements shown in Figure 1, we show that our observation that the solubility of CO_2 decrease with functionality still holds.

At 60 °C, the measurements in Figure 3 of the main text show that Henry's constant either stays constant or decreases with molecular weight between 400 g/mol and 1000 g/mol. Therefore, our observation that the solubility of CO_2 decreases with functionality at 60 °C is also still true after accounting for the discrepancy in the molecular weight.

4 Distinguishing Effects of Dissolved CO_2 Fraction and Temperature on Interfacial Tension

In Figure 4b of the main text (measured data are shown for convenience in Figure S6a), the interfacial tension between the polyol-rich and CO_2-rich phases decreases with pressure, but the effect of temperature depends on the pressure. At low pressure, interfacial tension increases with temperature, whereas at high pressure, interfacial tension decreases with temperature. The reason for this nontrivial effect is more clearly seen in Figure S6b, where the interfacial tension is plotted as a function of the weight fraction of dissolved CO_2. Here, increasing the temperature decreases the interfacial tension, as is observed for pure liquids due to the increased entropic driving force for mixing. At a fixed pressure, however, increasing the temperature decreases the amount of dissolved CO_2, and the amount by which

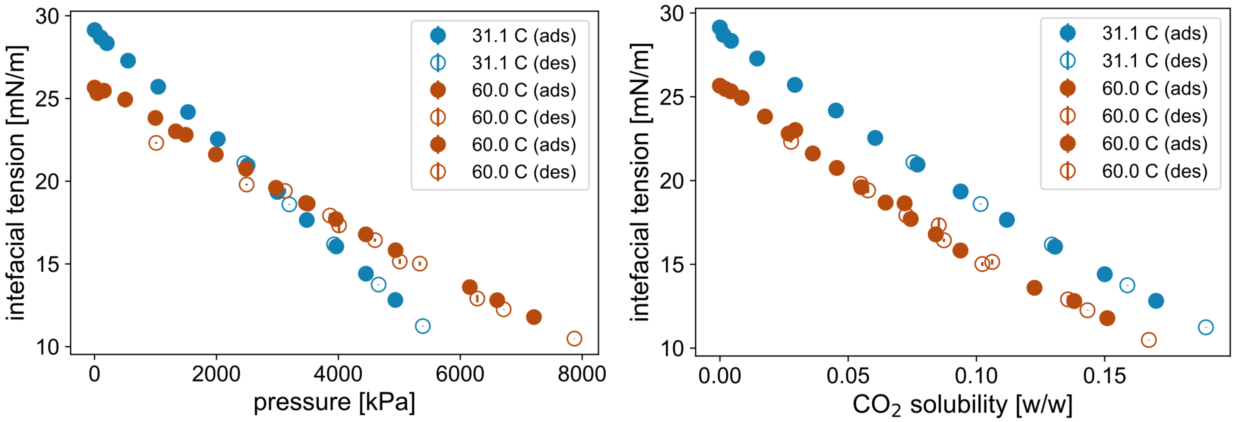

(a) Interfacial tension vs. pressure

(b) Interfacial tension vs. solubility

Figure S6: Interfacial tension of a mixture of PPG and CO_2 between the polyol-rich and CO_2-rich phases G-ADSA. Measurements were taken during both pressurization (filled circles) and depressurization (empty circles) of CO_2 with agreement within uncertainty. Error bars shown but may be smaller than glyph for some data points. Data are shown at 31 °C (blue) and 60 °C (red).

it decreases is greater at higher pressure (see Figure S8a). Because the interfacial tension decreases with the amount of dissolved CO_2 (due to the increased similarity of the polyol–CO_2 mixture to the CO_2 atmosphere), the reduction in dissolved CO_2 with temperature can increase the interfacial tension more than raising the temperature alone decreases it. The result is the increase in the interfacial tension with temperature at pressures above 3000 kPa. To our knowledge, measurements distinctly demonstrating this crossover in the interfacial tension with temperature have not been reported in the literature.

5 Sensitivity of PC-SAFT and DFT Models to Variations in Parameters

In the main text, a PC-SAFT model was used to model the solubility and specific volume of the polyol–CO_2 mixture. While CO_2 parameters were obtained from the literature based on the CO_2 equation of state, parameters for the polyol (PPG 2700 g/mol) were selected to match the measurements of the solubility of CO_2. These parameters are reported in Table

2 of the main text. Because we fit these parameters through trial and error, we did not compute confidence intervals. Instead, we show the effect of ±5% variations in each of these parameters on the predictions of different properties made by the PC-SAFT (for specific volume and solubility) and corresponding DFT (for interfacial tension) models in Figure S7.

The properties of the polyol–CO_2 mixture are most sensitive to the polyol bead size σ, as seen in the left column of Figure S7. CO_2 solubility increases with bead size while interfacial tension decreases, in large part due to the increased concentration of dissolved CO_2. The specific volume increases with bead size since the molecular weight of each bead is kept the same, but interestingly the effect of pressure on the specific volume reverses. For small bead size ($\sigma = 2.86$ Å, blue line), the specific volume increases with pressure, while at large bead size ($\sigma = 3.16$ Å, red line), the specific volume decreases with pressure. Interestingly, the best quantitative agreement with the G-ADSA measurements is achieved with the large bead size, while the best qualitative agreement with the general increase in specific volume with pressure is achieved with the small bead size. We expect that a different model is needed to achieve both quantitative and qualitative agreement, likely one that accounts for the association interactions of hydrogen bonding based on the recommendation of Parks and Beckman[S6].

The effect of the polyol bead interaction strength ϵ on the polyol–CO_2 properties is smaller and opposite the effect of σ, as seen in the center column of Figure S7. Because their effects are opposite, ϵ can be varied to "cancel out" the effect of varying σ, such that the same predictions for solubility and interfacial tension are made for a degenerate set of pairs of σ and ϵ. The effect on specific volume is not entirely opposite, however, because varying ϵ has a negligible effect on the qualitative behavior of the specific volume with pressure and the quantitative effect of varying σ is significantly more than that of varying ϵ. Consequently, within this degenerate set of pairs of σ and ϵ, the predictions for specific volume can be adjusted while those for solubility and interfacial tension remain the same.

Finally, the effect of the molecular weight is negligible on the scale of 5% variations, as

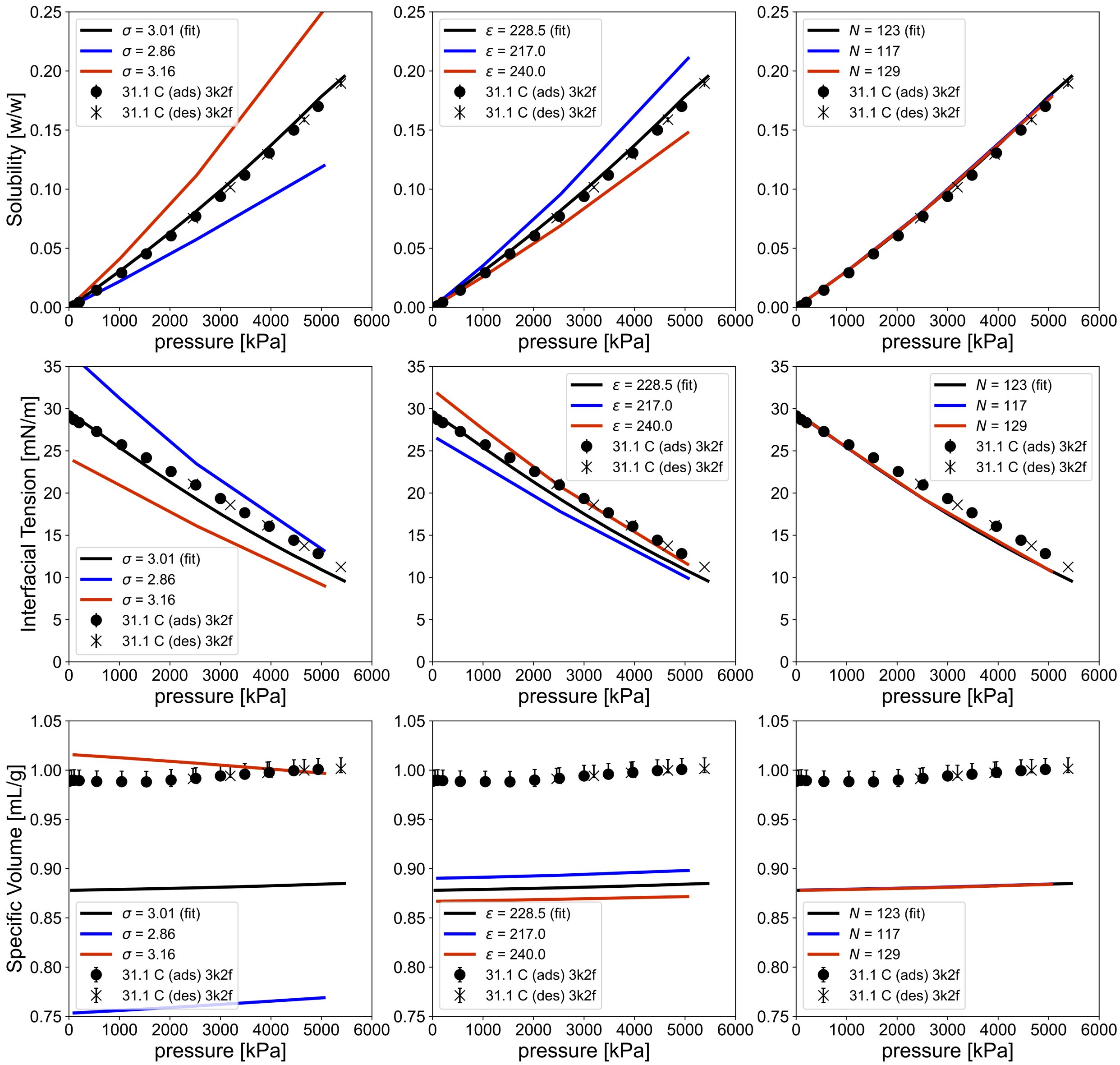

Figure S7: The sensitivity of the predictions of our PC-SAFT and DFT models to the polyol parameters σ (bead size, Å, first column), ε (energy parameter, k_B, second column), and N (number of beads, third column) to $+5\%$ (red) and -5% (blue) variations about the fitted value (black) are plotted for the solubility of CO_2 (first row, PC-SAFT), interfacial tension (second row, DFT), and specific volume (third row, PC-SAFT) of a mixture of CO_2 and PPG (2700 g/mol). The G-ADSA data measured at 31.1 °C are shown in black (adsorption ○ and desorption ×). Error bars may be smaller than glyphs for some data points.

seen in the far right column of Figure S7. The model predictions for each value of N are almost indistinguishable at the scale at which they are shown. We believe that the reason for the small size of the effect of N is that its primary contribution to the free energy comes

into the translational entropy of the polymer, which is proportional to $1/N$. At $N = 123$, a 5% change in N yield a roughly 5% change in $1/N$, which is already small. We expect that a significant decrease in N would increase the translational entropy of the polymer and drive greater mixing, *i.e.*, greater solubility of CO_2 given that the CO_2-phobicity of the hydroxyl end groups is not accounted for in this model.

5.1 Alternative Parameters

Given that the PC-SAFT model underestimated the specific volume (see Figure 4c in the main text), we explored other model parameters that deviated more from the predictions of the group contribution method.[S7] We found that by increasing both polyol bead size σ_{polyol} and the polyol interaction energy ϵ_{polyol} in the appropriate proportion, we could increase the estimate for the specific volume to be closer to the experimental measurement with negligible effect on the estimates for solubility or interfacial tension, as shown in Figure S8a,b. Nevertheless, with these larger parameters, while our model's predictions of the specific volume of the polyol-rich phase were more *quantitatively* accurate, our model predicted that the specific volume would decrease with pressure (Figure S8c), whereas our measurements showed that the specific volume generally increases with pressure (likely due to the greater absorption of CO_2, which has a higher specific volume than polyol).

In general, SAFT-type equations are prone to degeneracies in the model parameters, particularly when there is a lack of experimental data to fit them to[S8,S9]. Within this work, we use the root-mean-squared-error (RMSE) as the objective function ($f_{\text{obj.}}$) for fitting the polyol parameters in the PC-SAFT equation:

$$f_{\text{obj.}} = \sum_{i}^{N_{\text{set}}} \sqrt{\frac{1}{N_{\text{data},i}} \sum_{j} (X_{i,j}^{\text{pred.}} - X_{i,j}^{\text{exp.}})^2} \tag{9}$$

where N_{set} is the number of data sets used, $N_{\text{data},i}$ is the number of data points in set i, X is a particular property, and the superscripts "pred." and "exp." denote predictions and

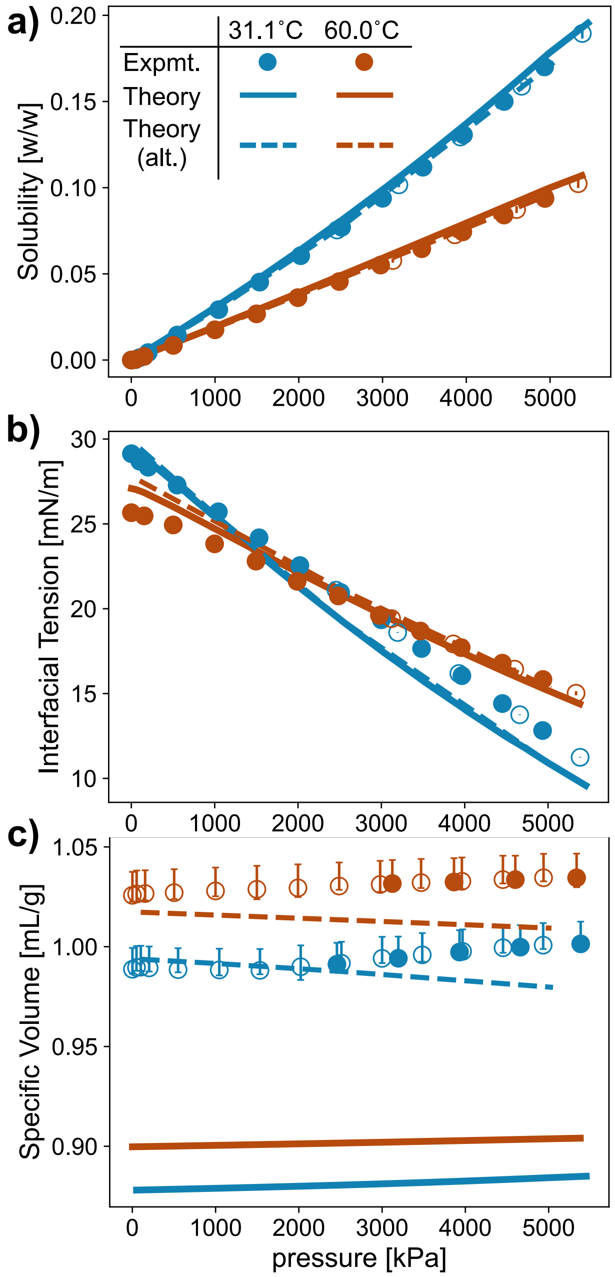

Figure S8: Comparison between the PC-SAFT model and measurements of a mixture of CO_2 and 2700 g/mol PPG is shown for two sets of PC-SAFT parameters. The predictions of the model using the parameters listed in Table 2 is plotted in solid lines. The predictions of the model using $\sigma = 3.17$ Å and $\epsilon = 253\ k_B$ for the polyol (same $N = 123$) are shown with dashed lines (alternative theory). Data are plotted as a function of pressure [kPa] for two temperatures, 31.1 °C (blue) and 60 ° (red). a) Solubility of CO_2 [w/w] in polyol. b) Interfacial tension between polyol-rich and CO_2-rich phases [mN/m]. c) Specific volume of polyol-rich phase.

experimental values of this property, respectively.

Typically, saturation pressure and saturated liquid densities for pure components are used to fit pure component parameters[S9], which usually results in a set of non-degenerate parameters; however, in this case, only the saturated specific volumes and CO_2 solubilities at various pressures and temperature were available to fit the polyol parameters. As a result, the polyol parameters are degenerate. We show this degeneracy by plotting a heat map of this objective function $f_{\text{obj.}}$ as a function of different PC-SAFT parameters (number of beads $N_{2.7k}$, bead size σ, and energy parameter ϵ) of the 2700 g/mol PPG polyol in Figure S9. Note that for better visualization, we have fixed the k_{ij} parameter to be 0, so the optima in Figure S9 do not correspond exactly to the optimal parameters discussed in the text; however, the qualitative trends will be similar.

When the present formulation of the PC-SAFT equation is fit to only the CO_2 solubility data (Figures S9a and S9c), the parameters are highly degenerate, meaning that the difference in the value of the objective function is minute over a wide range of parameter values (extended dark blue region, indicating a wide range for which the objective function is near zero). For example, the effect of the number of beads (Figure S9a) is negligible over a range of $\pm 10\%$, consistent with Figure S7 (see rightmost column)). Furthermore, as shown in Figure S9c, the bead size and energy parameters are also highly degenerate when varied together, as indicated by the slant of the degenerate region (dark blue). Consistent with the conclusions in the previous Section, increasing the value of both the bead size σ and energy parameter ϵ can result in a similar value of the objective function. Interestingly, based on S9c, one can also conclude that increasing the bead size but decreasing the energy parameter could result in a similar value of the objective function. However, based on Figure S7, we can anticipate that this will worsen our predictions for the CO_2 solubility and interfacial tension, so we do not expect the parameters in this degeneracy region to result in accurate estimates when k_{ij} is optimized as well.

The degeneracy in the polyol parameters can be "broken" by fitting the PC-SAFT pre-

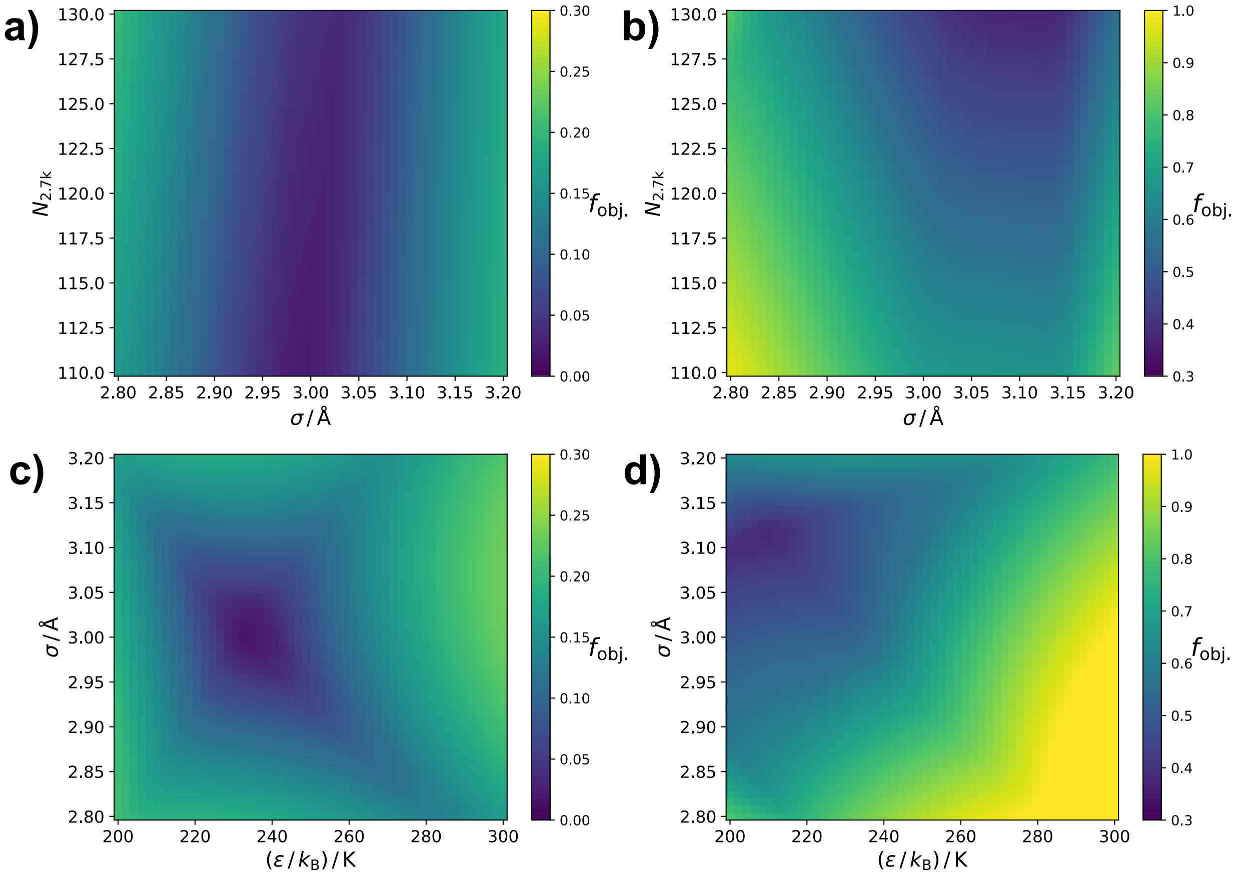

Figure S9: Heat map of the objective function, $f_{\text{obj.}}$: RMSE using only CO_2 solubility data (a and c) or CO_2 solubility and specific volume data (b and d). The axes represent the variation of the PC-SAFT parameters of the polyol: $N_{2.7\text{k}}$ (number of beads for 2700 g/mol polyol), σ (bead size, Angstroms), ϵ (energy parameter, Boltzmann's constant k_{B}). All other parameters are kept constant at their values in the original theory (see Table 2 in the main text) except for k_{ij}, which was set to 0 for simplicity. Generated using Clapeyron.jl[S10].

dictions to both the CO_2 solubility and the specific volume, as shown in Figures S9b and S9d. Breaking this degeneracy comes at the cost of a higher value of the objective function, however, which is the reason that the specific volume cannot be modeled accurately by the present formulation of PC-SAFT (as discussed in Section 4.3 of the main text). The breaking of this degeneracy is seen by the presence of clear minima (compact dark blue regions indicating the where the object function is lowest) in Figures S9b and S9d. These minima are quite broad, however, particularly in Figure S9b where, for a constant number of beads, the bead size can vary by a significant amount while leaving the objective function relatively

unchanged. Nevertheless, consistent with Figure S8, a large bead size should result in better predictions for the specific volume. Interestingly, Figure S9d would seem to indicate that a lower value in the energy parameter should result in a better fit. However, it is important to bear in mind that, based on Figure S7, we are aware that this would worsen the predictions of the CO_2 solubility and, in Figure S9d, k_{ij} is kept constant (explaining why the optimum is not located at the parameters given in figure S7). To optimize the objective with respect to k_{ij} as well, the random search algorithm provided by the BlackBoxOptim.jl[S11] package can be used with Clapeyron.jl[S10]. This more comprehensive fit recovers the parameters given in Table 2 of the main text and Figure S8 when using either only the CO_2 solubility data or all of the available data, respectively.

In addition, considering that the magnitude of the objective function in Figure S9 when including the specific volume data is greater than when only CO_2 solubility data is included, and that it is not possible to qualitatively reproduce the trends in specific volume with respect to pressure (Figure S8), one could conclude that the reason for these deficiencies is due to association interactions not being included in the model. The inclusion of association between polyol and carbon dioxide would result in a lower value of the objective function and would be consistent with previous works[S6]. The difficulty of including association within a SAFT-type equation is that the parameters needed are highly degenerate, requiring additional experimental data to "break" these degeneracies.[S12] Thus, while incorporating association interactions would improve the qualitative and quantitative accuracy of the present formulation, it is unlikely that the data available are sufficient to fit the additional parameters. Furthermore, the DFT approach used in this work would need to be reformulated to incorporate the effects of association, which has not been reported in the literature to our knowledge.

6 DFT Predicts Non-monotonic CO_2 Concentration Profile

In the main text, we only discuss the interfacial tension along the vapor–liquid interface predicted by our DFT model. This interfacial tension is computed from a complete density profile along the interface. An example of such a density profile predicted by our DFT model is shown in Figure S10. The plot shows the concentrations of CO_2 and PPG (2700 g/mol) at the interface between the CO_2-rich (left) and polyol-rich (right) phases. Interestingly, the CO_2 partitions similarly in both phases but accumulates at the interface. Such behavior is reminiscent of a surfactant and suggests that CO_2 may condense at the surface of bubbles that form at high pressure to mediate the drastic change in density from polyol-rich liquid to CO_2-rich vapor. This non-monotonic density profile of the volatile component was predicted with DFT for a binary mixture of liquid and gas by Talanquer and Oxtoby[S13] as well as for a binary mixture of CO_2 and PMMA[S14–S16] and CO_2 and P(MMA-*co*-EA) copolymer.[S17] Talanquer and Oxtoby attribute the decrease in the interfacial tension with pressure predicted by DFT (see Fig. 4b) to the increase in the amount of this enrichment of the volatile component along the liquid–vapor interface.

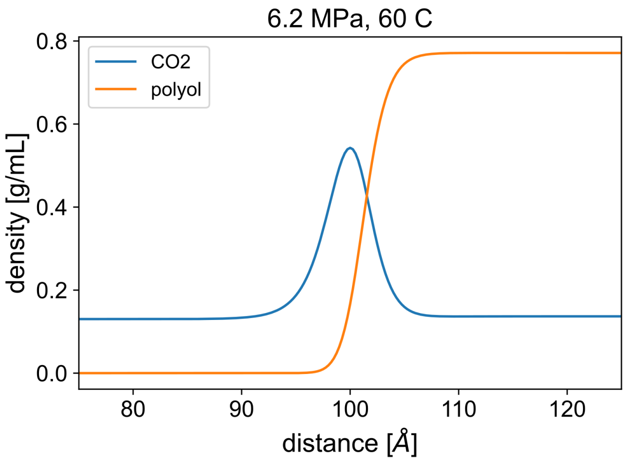

Figure S10: Density profiles of CO_2 and PPG (2700 g/mol) at the interface between CO_2-rich (left, $<$ 100 Å) and polyol-rich (right, $>$ 100 Å) phases at 6.2 MPa and 60 °C. Note that CO_2 has a similar partitioning in both phases but accumulates at the interface.

References

(S1) Ylitalo, A. S. andylitalo/g-adsa: v0.1.0. 2022; https://github.com/andylitalo/g-adsa.

(S2) NIST, NIST Standard Reference Database Number 69. 2022; https://webbook.nist.gov/chemistry/.

(S3) Pastore Carbone, M. G.; Di Maio, E.; Iannace, S.; Mensitieri, G. Simultaneous experimental evaluation of solubility, diffusivity, interfacial tension and specific volume of polymer/gas solutions. Polym. Test. **2011**, 30, 303–309, DOI: 10.1016/j.polymertesting.2011.01.004.

(S4) Song, B.; Springer, J. Determination of interfacial tension from the profile of a pendant drop using computer-aided image processing. J. Colloid Interface Sci. **1996**, 184, 64–76, DOI: 10.1006/jcis.1996.0597.

(S5) Ylitalo, A. S. A Bubble is Born: Nucleation and Early Growth of CO2 Bubbles in Polymer Foams. Ph.D. thesis, California Institute of Technology, 2022.

(S6) Parks, K. L.; Beckman, E. J. Generation of microcellular polyurethane foams via polymerization in carbon dioxide. I: Phase behavior of polyurethane precursors. Polym. Eng. Sci. **1996**, 36, 2404–2416, DOI: 10.1002/pen.10639.

(S7) Tihic, A.; Kontogeorgis, G. M.; von Solms, N.; Michelsen, M. L.; Constantinou, L. A Predictive Group-Contribution Simplified PC-SAFT Equation of State: Application to Polymer Systems. Ind. Eng. Chem. Res. **2008**, 47, 5092–5101, DOI: 10.1021/ie0710768.

(S8) de Villiers, A. J.; Schwarz, C. E.; Burger, A. J.; Kontogeorgis, G. M. Evaluation of the PC-SAFT, SAFT and CPA equations of state in predicting derivative properties

of selected non-polar and hydrogen-bonding compounds. Fluid Phase Equilib. **2013**, 338, 1–15, DOI: 10.1016/j.fluid.2012.09.035.

(S9) Dufal, S.; Lafitte, T.; Galindo, A.; Jackson, G.; Haslam, A. J. Developing intermolecular-potential models for use with the SAFT-VRMie equation of state. AIChE J. **2015**, 61, 2891–2912, DOI: 10.1002/aic.14808.

(S10) Walker, P. J.; Yew, H.-W.; Riedemann, A. Clapeyron.jl: An Extensible, Open-Source Fluid Thermodynamics Toolkit. Ind. Eng. Chem. Res. **2022**, 61, 7130–7153, DOI: doi/10.1021/acs.iecr.2c00326.

(S11) Feldt, R.; Contributors, BlackBoxOptim.jl. 2022; https://github.com/robertfeldt/BlackBoxOptim.jl.

(S12) Dufal, S.; Lafitte, T.; Haslam, A. J.; Galindo, A.; Clark, G. N.; Vega, C.; Jackson, G. The A in SAFT: developing the contribution of association to the Helmholtz free energy within a Wertheim TPT1 treatment of generic Mie fluids. Mol. Phys. **2015**, 113, 948–984, DOI: 10.1080/00268976.2015.1029027.

(S13) Talanquer, V.; Oxtoby, D. W. Nucleation of bubbles in binary fluids. J. Chem. Phys. **1995**, 102, 2156–2164, DOI: 10.1063/1.468737.

(S14) Xu, X.; Cristancho, D. E.; Costeux, S.; Wang, Z.-G. Bubble nucleation in polymer–CO2 mixtures. Soft Matter **2013**, 9, 9675, DOI: 10.1039/c3sm51477c.

(S15) Xu, X.; Cristancho, D. E.; Costeux, S.; Wang, Z.-G. Density-functional theory for polymer-carbon dioxide mixtures: A perturbed-chain SAFT approach. J. Chem. Phys. **2012**, 137, 054902, DOI: 10.1063/1.4742346.

(S16) Xu, X.; Cristancho, D. E.; Costeux, S.; Wang, Z.-G. Discontinuous Bubble Nucleation Due to a Metastable Condensation Transition in Polymer–CO2 Mixtures. J. Phys. Chem. Lett. **2013**, 4, 1639–1643, DOI: 10.1021/jz4005575.

(S17) Xu, X.; Cristancho, D. E.; Costeux, S.; Wang, Z.-G. Density-Functional Theory for Mixtures of AB Random Copolymer and CO 2. Macromolecules **2015**, 48, 6035–6046, DOI: 10.1021/acs.macromol.5b01122.
